# Eribulin normalizes pancreatic cancer-associated fibroblasts by simulating selected features of TGFβ inhibition

**DOI:** 10.1186/s12885-022-10330-y

**Published:** 2022-12-02

**Authors:** Tiffany Luong, Edna Cukierman

**Affiliations:** Cancer Signaling and Microenvironment, Marvin and Concetta Greenberg Pancreatic Cancer Institute, Fox Chase Cancer Center, Temple Health, Philadelphia, PA 19111 USA

**Keywords:** Eribulin, TGFβ, Extracellular matrix, cancer-associated fibroblasts, Cell-derived ECM, Pancreatic cancer

## Abstract

**Background:**

Less than 11% of pancreatic cancer patients survive 5-years post-diagnosis. The unique biology of pancreatic cancer includes a significant expansion of its desmoplastic tumor microenvironment, wherein cancer-associated fibroblasts (CAFs) and their self-produced extracellular matrix are key components. CAF functions are both tumor-supportive and tumor-suppressive, while normal fibroblastic cells are solely tumor-suppressive. Knowing that CAF-eliminating drugs are ineffective and can accelerate cancer progression, therapies that “normalize” CAF function are highly pursued. Eribulin is a well-tolerated anti-microtubule drug used to treat a plethora of neoplasias, including advanced/metastatic cancers. Importantly, eribulin can inhibit epithelial to mesenchymal transition via a mechanism akin to blocking pathways induced by transforming growth factor-beta (TGFβ). Notably, canonical TGFβ signaling also plays a pivotal role in CAF activation, which is necessary for the development and maintenance of desmoplasia. Hence, we hypothesized that eribulin could modulate, and perhaps “normalize” CAF function.

**Methods:**

To test this premise, we used a well-established in vivo-mimetic fibroblastic cell-derived extracellular matrix (CDM) system and gauged the effects of eribulin on human pancreatic CAFs and cancer cells. This pathophysiologic fibroblast/matrix functional unit was also used to query eribulin effects on CDM-regulated pancreatic cancer cell survival and invasive spread.

**Results:**

Demonstrated that intact CAF CDMs modestly restricted eribulin from obstructing pancreatic cancer cell growth. Nonetheless, eribulin-treated CAFs generated CDMs that limited nutrient-deprived pancreatic cancer cell survival, similar to reported tumor-suppressive CDMs generated by TGFβ-deficient CAFs.

**Conclusions:**

Data from this study support the central proposed premise suggesting that eribulin could be used as a CAF/matrix-normalizing drug.

**Supplementary Information:**

The online version contains supplementary material available at 10.1186/s12885-022-10330-y.

## Background

Pancreatic ductal adenocarcinoma (PDAC) is a devastating disease with limited therapeutic options [1]. This disease is estimated to become the second leading cause of cancer-related deaths in the United States by 2026 [2]. Currently, less than 11% of patients diagnosed with PDAC are estimated to survive up to 5-years [3]. Thus, it is of utmost importance to identify novel effective therapies for treating patients diagnosed with PDAC.

One of the major features of PDAC is its abundant desmoplastic stroma, characterized by an expansion of activated resident fibroblasts and the deposition of an interstitial extracellular matrix (ECM) by these cells, generating a fibroblastic/ECM functional unit. Since desmoplasia can encompass over 70% of the tumor mass, this unique tumor microenvironment (TME) plays a major role in the manner drugs influence disease progression [4–6]. Although the TME is known to modulate PDAC onset and progression, ablating it can accelerate tumorigenesis [7–9]. Consequently, efforts to prevent eroding the TME, while modulating it to retain a tumor-suppressive stroma (also known as “stroma normalization”), are highly sought [6, 10–14]. A possible approach for achieving cancer-associated fibroblast (CAF) “normalization” is to revert CAF/ECM units to their original tumor-suppressive state. This could potentially be done by limiting signaling induced by desmoplastic tumor-promoting factors such as the transforming growth factor-beta (TGFβ). This is because ligands from the TGFβ family are known to drive CAF/ECM unit activation [10]. Further, high levels of phosphorylated SMAD2/3 (pSMAD2/3; Small Worm Phenotype and Mothers Against Decapentaplegic 2 and 3) in human CAFs, which are indicative of constitutively high canonical TGFβ activity in these functional units, have been shown to inversely correlate with PDAC and renal cancer overall survival [15]. Unfortunately, directly targeting TGFβ has not yet been clinically efficacious [16]. Hence, drugs that at minimum, partially simulate TGFβ inhibition, and demonstrate high patient tolerability (e.g., imparting low toxicity), could serve as valuable alternative therapeutics for targeting CAF/ECM units and attain tumor suppressive TME normalization.

Eribulin is an anti-microtubule agent that was approved in 2010 by the Food and Drug Administration to treat metastatic breast cancer [17, 18]. A mechanism often used by metastatic cells that renders cells with stem-like properties, is the TGFβ-modulated epithelial-to-mesenchymal transition (EMT [19]). Of note, eribulin was reported to revert metastatic EMT through a mechanism that dampens pSMAD2/3 levels [20]. Further, drugs known to prevent EMT, as well as drugs that block or revert mesenchymal cell activation [21], constitute potential stroma-normalizing agents [10, 13–15, 22–26]. Hence, we hypothesized that treating CAF/ECM units with eribulin could, at least in part, simulate TGFβ-signaling blockage and render a “normalized” (e.g., tumor-suppressive) microenvironment.

To this end, our team previously developed an in vivo-like fibroblastic three-dimensional (3D) cell culturing system, which effectively reproduces the pathophysiology of the CAF/ECM functional unit [27, 28]. In fact, this system has been vetted in vivo using both murine models and human samples and our team has adapted it for the use of PDAC patient-harvested CAFs [15, 22, 23, 29]. Further, the system has been successfully used for assessing how drugs modulate CAF function, as well as how drug effects are altered by fibroblastic ECMs [28, 30, 31]. Hence, our CAF-generated cell-derived ECM system, also known as “the CDM system,” was used in this study for conducting a series of tests that were designed to measure the effects of eribulin in modulating CAF/ECM unit phenotypes and functions. Further, since pancreas resident fibroblastic cells, initially presenting with normal-like tumor-suppressive features, undergo an ex-vivo culturing stress-induced activation [32], our team previously used the CRISPR/Cas9 system to engineer αvβ5-integrin deficient PDAC CAFs (β5^KO^) [15]. These β5^KO^ CAFs perpetuate a phenotype that simulates TGFβ inhibition in CAFs [15] and constituted an important control-model used in this study as a “normalized” CAF/ECM unit control [22, 33].

We herein report that CAF-generated ECMs somewhat protect PDAC cells by hindering the effects of eribulin to limit tumor cell growth. Importantly, we also report that eribulin can nonetheless act on CAF/ECMs and prompt these functional units to generate an ECM that no longer effectively promotes PDAC cell invasive spread and that has lost its ability to nurture PDAC cells under nutrient-limited conditions. Of note, the observed CAF-generated ECM functional shift, from tumor-supportive to tumor-suppressive, simulated reported results that were obtained with TGFβ deficient CAFs in our CDM system [15, 22, 33]. In addition, we report that eribulin promoted CAF/ECM units to limit pro-tumorigenic IL6 cytokine secretion. Hence, this study proposes that eribulin could be used as a stroma-normalizing agent as it simulates some key features known to be imparted by the inhibition of TGFβ signaling in CAFs.

## Methods

### Ethics statement informing on the previously collected tissue used to harvest the human fibroblastic cells utilized

All fibroblastic human cells used in this study, were harvested from surgical patient samples in accordance with guidelines and regulations at Fox Chase Cancer Center following institutional biosafety policies. Fresh surgical samples were collected by the Biosample Repository Facility (BRF) at Fox Chase Cancer Center. Prior to surgery, patients interested in donating tissue for research are informed on how the tissue is collected, processed, and distributed, as well as the type of data and decoding process that is undertaken by the BRF personnel. If patients agree, they proceed to sign a written consent that was approved by the Institutional Review Board (IRB) protocol at Fox Chase. To assure protecting the patient identities and specifically for this study, solely the BRF-generated specimen number and organ of origin were conveyed to the research team. All cells used in this study were harvested from surgical samples that were obtained from patients who signed a consent allowing to decode their samples and use the anonymized materials for research according to the approved IRB protocol at Fox Chase. Hence, while no human subjects were used in this study the authors confirm that the experimental protocols were approved by the corresponding Fox Chase IBC, IRB and licensing committees.

### Cells and culturing conditions

Human PDAC CAF lines were generated from two independent PDAC surgical samples. Using the parental CAF line from one of these patients, a GFP-CRISPR^KO^ control was also used and it is referred to as CAF’ throughout the text. CAF’ served as a control for the αvβ5-integrin deficient CAF line (β5^KO^), which was engineered from the same parental CAF line. Of note, β5^KO^ CAFs are known to sustain normal-like traits and functions. This is because these normalized CAFs cannot be activated in the absence of a mature TGFβ1 ligand [15]. A matching tumor-adjacent fibroblastic CAF line maintaining normal-like traits and functions, and harvested from a benign PDAC adjacent tissue, was used in the re-plating experiments designed to test the ability of CAF-generated ECMs to induce a normal-to-CAF activation overnight, as published [15, 23]. CAFs harvested by a second PDAC surgical sample are referred to as “CAF 2” throughout the text. Additionally, a human lung CAF line was used for added rigor. All fibroblastic cells were isolated from fresh surgical tissue collected at Fox Chase Cancer Center (Philadelphia, PA) using an enzymatic tissue digestion approach, and immortalized with hTERT as published [28]. Fibroblasts were authenticated and tested for mycoplasma as previously reported [15, 23]. Panc-1 human PDAC cells were purchased from the American Type Culture Collection (Manassas, VA) and used within 6 months of thawing. All cells were cultured in high-glucose Dulbecco’s Modified Eagle Medium (DMEM) supplemented with 10% Fetal Bovine Serum (FBS, Peak Serum, Wellington, CO), 2 mM L-glutamine, and 100 U/mL-µg/mL and penicillin-streptomycin (Corning, Manassas, VA) at 37 °C with 5% CO_2_ unless otherwise specified.

### Chemicals or drugs

Eribulin-mesylate was provided as a lyophilized powder by Eisai Inc. (Woodcliff Lake, NJ). Lyophilized powder was reconstituted in HPLC grade DMSO to make 10 mM stock solutions. Paclitaxel was purchased from Sigma-Aldrich (St. Louis, MO) and reconstituted in DMSO to make 1.17 mM stock solutions. Stock solutions were aliquoted for single-use thawing, stored at -80 °C, and diluted with cell culture medium to achieve the desired experimental concentrations. SB431542 hydrate, a selective inhibitor of TGFβ Type 1 receptor kinases was purchased from Sigma-Aldrich (St. Louis, MO) and used at a concentration of 10 µM in all experiments. A recombinant/matured form of transforming Growth Factor-β1 was also purchased from Sigma-Aldrich and used at 1 ng/mL or 10 ng/mL.

### Viability assay (2D)

Panc-1 or CAFs were seeded at a density of 2 × 10^3^ cells per well in a 96-well plate, overnight. The next day, cells were treated with either eribulin, paclitaxel, SB431542, or DMSO vehicle daily for 5 days at the indicated concentrations. Alamar blue (Thermo Fisher Scientific, Waltham, MA) was added on the 6th day and incubated at 37 °C, 5% CO_2_ for 4 h. Cell viability was measured using Tecan Spark™ 10 M microplate reader (Tecan, Switzerland) at an absorbance of 570 nm and a reference wavelength of 600 nm according to the manufacturer’s protocol.

### Fibroblastic cell-derived ECM (CDM) system

Fibroblastic cell-derived ECMs (known as CDMs; to attain fibroblastic/ECM functional units in vitro) and decellularized CDMs were generated as previously published [28, 34]. The CDM system was used to measure ECM production in the presence of eribulin or drug controls (e.g., paclitaxel, SB431542, or DMSO vehicle) at the concentrations stated in the text. Using the same CDM system, β5^KO^ CAFs were used to measure ECM production in the presence of the mature form of TGFβ1 at 1 ng/mL or 10 ng/mL with the addition of eribulin, SB431542 or DMSO control. Briefly, confluent fibroblastic cultures were supplemented with 50 µg/mL of ascorbic acid daily for a total of 5 days [28, 34]. The quality of ECM production was confirmed via indirect immunofluorescence (see method below). The thickness of ECM, indicative of qualified matrix production, was measured to reach a minimum of 7 μm; ECMs thinner than this were not considered of high quality and were hence excluded from all analyses. For re-plating and 3D survival assays (see method below), fibroblastic/ECM units were decellularized, to attain acellular CDMs, by treating cultures with PBS containing 0.5% Triton X-100 and adding 20 mM NH_4_OH (Sigma-Aldrich, St. Louis, MO) at the end of ECM production. The resulting decellularized CDMs were treated with DNase I and washed with PBS before cellular re-inoculation, as published [28]. Eventually, tumor-adjacent fibroblasts or PDAC cells were re-plated.

### Normal-to-CAF functional transition: re-plating assay

Decellularized drug or vehicle-treated CAF-generated CDMs were used to re-plate tumor-adjacent fibroblastic cells, obtained from a PDAC adjacent tissue, matching one of the PDAC CAFs [15]. 7 × 10^3^ fibroblastic cells, from the above described adjacent tissue, vetted first to sustain normal-like traits [28, 34], were seeded onto the assorted CAF-generated CDMs and cultured overnight. Levels of αSMA co-localization with stressed fibers (F-actin, phalloidin staining) were measured, as these are indicative of an effective ECM prompted normal-to-CAF fibroblastic cell transition [15, 27]. Cultures were stained with αSMA at 1:300 (100 µg/mL) and phalloidin 1:40 (3.3 µM) via indirect immunofluorescence (see method below). Images were acquired with a plan Apo λ 60x oil immersion objective using a Nikon A1S confocal system. Monochromatic image stacks were reconstituted in ImageJ (NIH, Bethesda, MD) and the percentage levels of co-localization of αSMA to stressed fibers (F-actin, phalloidin stain) was assessed using the MetaMorph 7.8.0.0 software.

### Survival assay (3D)

Survival assay was performed similarly to what was published by Drs. Francescone, R., and Vendramini-Costa D.B., et al. [23]. Briefly, 1.5 × 10^3^ red fluorescent protein (RFP)-expressing Panc-1 cells were seeded onto assorted decellularized CDMs under nutrient-depleted conditions (i.e., serum-free, L-glutamine-free media; unless otherwise stated), in the presence or absence of eribulin and compared with assorted drug and vehicle controls. The numbers of cells were measured/acquired every 24 h, up to 120 h, using a Nikon A1 confocal system equipped with a 10x (plan Apo) non-oil objective. Cell numbers were gauged as percentage red-cell area coverages per image with a minimum of 5 images acquired per sample.

### Cancer cell invasion spread assay

ECM invasion spread assays were conducted as previously published [35]. Briefly, 3 × 10^3^ Panc-1 cells per 30 µl were pre-labeled with Hoechst 33,342 (Thermo Fisher Scientific; Waltham, MA) for 1 h prior to trypsinization and suspended in tumor sphere-forming media (Irvine Scientific; Santa Ana, CA) supplemented with 2 U/mL of heparin and 0.5 µg/mL hydrocortisone. The individual 30 µl sample drops were carefully placed on a sterile 100 mm culture dish lid overnight with drops hanging upside down allowing spheroid formation. 5 ml of sterile PBS was added to the bottom of the dish to prevent hanging drops from drying out. Following 24 h, spheroids were carefully collected and deposited onto assorted decellularized CAF-generated CDMs, incubated for a few minutes to allow spheroids to adhere, then media was carefully added to the culture. Spheroids were incubated for 2 h to fully adhere, and this time was considered baseline (i.e., time 0). Images were acquired at baseline and again at 24 h for each spheroid using 4x magnification. For each condition, the spread of the spheroids was measured and reported as mean fold invasive spread (mean radius) per sphere. A minimum of 3 spheroids were recorded per experimental condition and experiments were repeated a minimum of two times per condition.

### Treatment of β5^KO^ CAF/ECM units with TGFβ

β5^KO^ CAFs were treated with recombinant/mature TGFβ1, at 1 ng/mL or 10 ng/mL, and DMSO or with eribulin at 1 nM during ECM production. On the third day of treatment, due to TGFβ1-induced myofibroblastic activation prompting for the collapse of β5^KO^ CAF/ECM units, the assay was ended for all experimental conditions. Images of the assorted fibroblastic cell/ECM units were acquired using the EVOS microscopy cell imaging system (Life Technologies, Waltham, MA); using the 10x and/or 4x objectives. This experiment was repeated two times with a minimum of three technical replicates per condition on each occasion.

### Indirect immunofluorescence

Samples were labeled by indirect immunofluorescence as published [27, 28]. Briefly, experimental samples were generated/cultured onto 7 or 12 mm coverslips (Ted Pella, Inc., Redding, CA and Carolina Biological, Burlington, NC, respectively). These were fixed and permeabilized in PBS supplemented with 4% paraformaldehyde (Electron Microscopy Sciences, Hatfield, PA), 5% sucrose (w/v), and 0.5% Triton X-100 for 3 min, followed by additional 20 min fixing with PBS containing 4% paraformaldehyde and 5% sucrose (w/v). Samples were blocked for a minimum of 1 h with Odyssey Blocking Buffer-PBS (LI-COR Biosciences, Lincoln, NE). The following primary antibodies were incubated for 90 min at room temperature using the antibodies and concentrations listed in Table 1.

**Table 1:** List of indirect immunofluorescence primary antibodies used

1° Antibody/reagent	Host	Concentration	Source	Catalog numbers
αSMA	Mouse	100 µg/mL	Sigma-Aldrich	A2547
Fibronectin	Rabbit	3 µg/µL	Sigma-Aldrich	F3648
anti-pFAK Y^397^	Rabbit	2.5 µg/µL	Invitrogen	44-624G
anti-human active α5β1 integrin	Mouse	45 µg/mL	SNAKA51, a gift from M Humphries [31]	N/A
DAPI	N/A	0.25 µg/µL	Invitrogen	D1306

Table depicting all antibodies used in immunofluorescence denoting the antigen, host, dilution, source, and catalog numbers

Samples were washed with PBS-Tween20 (0.05%) three times for 5 minutes each. Secondary donkey F(ab’) fragments [1:100] cross-linked to assorted fluorophores (Jackson ImmunoResearch Laboratories Inc., West Grove, PA), were incubated for 60 min at room temperature. Samples were washed as above and mounted using an anti-fade mounting reagent (Corning imaging recipe). Images were acquired with plan Apo λ 60x oil immersion objective using a Nikon A1S confocal system as above. Acquisition settings were kept identical for each primary antibody/fluorophore-liked F(ab’) secondary fragment. Sequential monochromatic images, corresponding to 0.5 µM thick slices, were reconstituted in ImageJ and processed for ECM or activated CAF biomarker evaluations as needed. A minimum of 5 images were acquired per sample. A minimum of 2 samples were prepared for each experiment, while experiments were repeated at least twice for each condition.

### Western blot

CDM-system generated cell cultures were lysed in RIPA buffer containing the following solutions: 150 mM sodium chloride, 1.0% Triton X-100, 0.5% sodium deoxycholate, 0.1% SDS (sodium dodecyl sulphate), and 50 mM Tris, pH 8.0. Protease and phosphatase inhibitors (Thermo Fisher Scientific, Waltham, MA) were added fresh before use. Cell lysates were resolved by SDS-PAGE (Sodium Dodecyl Sulfate PolyAcrylamide Gel Electrophoresis) using 4–20% precast gel (Biorad, Hercules, CA). Proteins were transferred by semi-dry transfer onto a PVDF (polyvinylidene fluoride) membrane. Membranes were blocked using 5% non-fat milk (Biorad, Hercules, CA) in TBST (0.01% Tween20) for a minimum of 1 h at room temperature. The following primary antibodies were incubated overnight at 4 °C as indicated in Table 2.

**Table 2:** List of western blot primary and secondary antibodies

1° Antibody	Host	Dilution (or Concentration)	Source	Catalog numbers
anti-palladin	Rabbit	0.0002 µg/µL	Protein tech, Chicago, IL	10853-1-AP
anti-phospho SMAD2 [S465/467]/SMAD3 [S423/425]	Rabbit	0.2 µg/mL	Cell Signaling, Danvers, MA	8828s
anti-SMAD2/3 (total)	Rabbit	0.2 µg/mL	Cell Signaling, Danvers, MA	3102s
Goat anti-mouse, IRDye^a^	Mouse	1:10,000	LI-COR, Lincoln, NE	926-68070
Goat anti-rabbit, IRDye^a^	Rabbit	1:10,000	LI-COR, Lincoln, NE	926-32211
Anti-mouse IgG, HRP-linked^b^	Mouse	1:5000	Cell Signaling, Danvers, MA	7076 S
Anti-Rabbit IgG, HRP-linked^b^	Rabbit	1:5000	Cell Signaling, Danvers, MA	7074 S

Table depicting all antibodies used denoting the antigen, host, dilution (or concentration), source, and catalog numbers

^a^indicate secondary antibodies used in Licor

^b^indicate secondary antibodies used in ECL

Following overnight incubations, membranes were washed 3 times with TBST for 15 min each. HRP-linked or Licor IRDye secondary antibodies were added and incubated for 1 h at room temperature. Membranes were again washed 3 times with TBST for 15 min each. Lastly, membranes using HRP-linked secondary were incubated with Immobilin Chemiluminescent HRP Substrate (ECL: Millipore, Burlington, MA) for 5 min before being developed onto films. Membranes using Licor IRDye secondary were read using Licor Odyssey imaging system (Lincoln, Nebraska).

### Assessment of lipid droplets

Fibroblastic/ECM units were cultured in the presence of eribulin or controls (DMSO vehicle, SB431542; see Methods on Fibroblastic cell-derived ECM (CDM) system above) and lipid droplets were assessed using the Lipid Droplets Fluorescence Assay Kit (Cayman Chemical, Ann Arbor, MI). Briefly, cells were washed with 0.1 mL of Assay Buffer and fixed with 0.05 ml of Fixative Solution for 10 min at room temperature. Cells were washed with Assay Buffer again and treated with 0.05 mL of the Nile Red Staining Solution (1:1000) along with Hoechst 33,342 (Thermo Fisher Scientific; Waltham, MA; 1:5000 at 20 mM stock) and incubated for 15 min at room temperature. The green fluorescence of Nile Red and blue fluorescent Hoechst channels were measured using the Tecan Spark™ 10 M microplate reader (Tecan, Switzerland) at 485 nm and 360 nm wavelengths respectively.

### ELISA (enzyme-Linked immunosorbent assay)

CAF/ECM unit-generated conditioned media were collected during the last 48 h of matrix production, when complete media (with serum) were replaced by serum-free media. Levels of secreted cytokines IL6, IL8, and TGFβ were measured via ELISA kits (R&D Systems, Minneapolis, MN) following manufacturer’s instructions. Reagents for IL6 and IL8 were obtained from DuoSet Ancillary Reagent Kit 2 (R&D Systems, Minneapolis, MN), and reagents for TGFβ were obtained from DuoSet Ancillary Reagent Kit 1 (R&D Systems, Minneapolis, MN). Briefly, 96 well microplates were coated overnight using the provided capture antibody for each cytokine. Wells were blocked for a minimum of 1 h at room temperature. Standards and samples were added and incubated for 2 h (IL6 and IL8 were diluted 1:250 and 100 µl samples were used for TGFβ). After 2 h–incubations, detection antibodies were incubate for an additional 2 h. Streptavidin-HRP was added to each well and incubate for 20 min. Substrate solution was added to each well and incubated for 20 min. Lastly, stop solution was added to each well and sample reactions were obtained using the Tecan Spark™ 10 M microplate reader (Tecan, Switzerland) set at 450 nm with reference wavelength set at 570 nm. Standard curves were generated using Graph Pad Prism 7.0 (San Diego, California). Values were normalized to the readout values corresponding to the Hoechst 33,342 (Thermo Fisher Scientific; Waltham, MA) channel.

### Statistical analysis

**Fig. 1:**
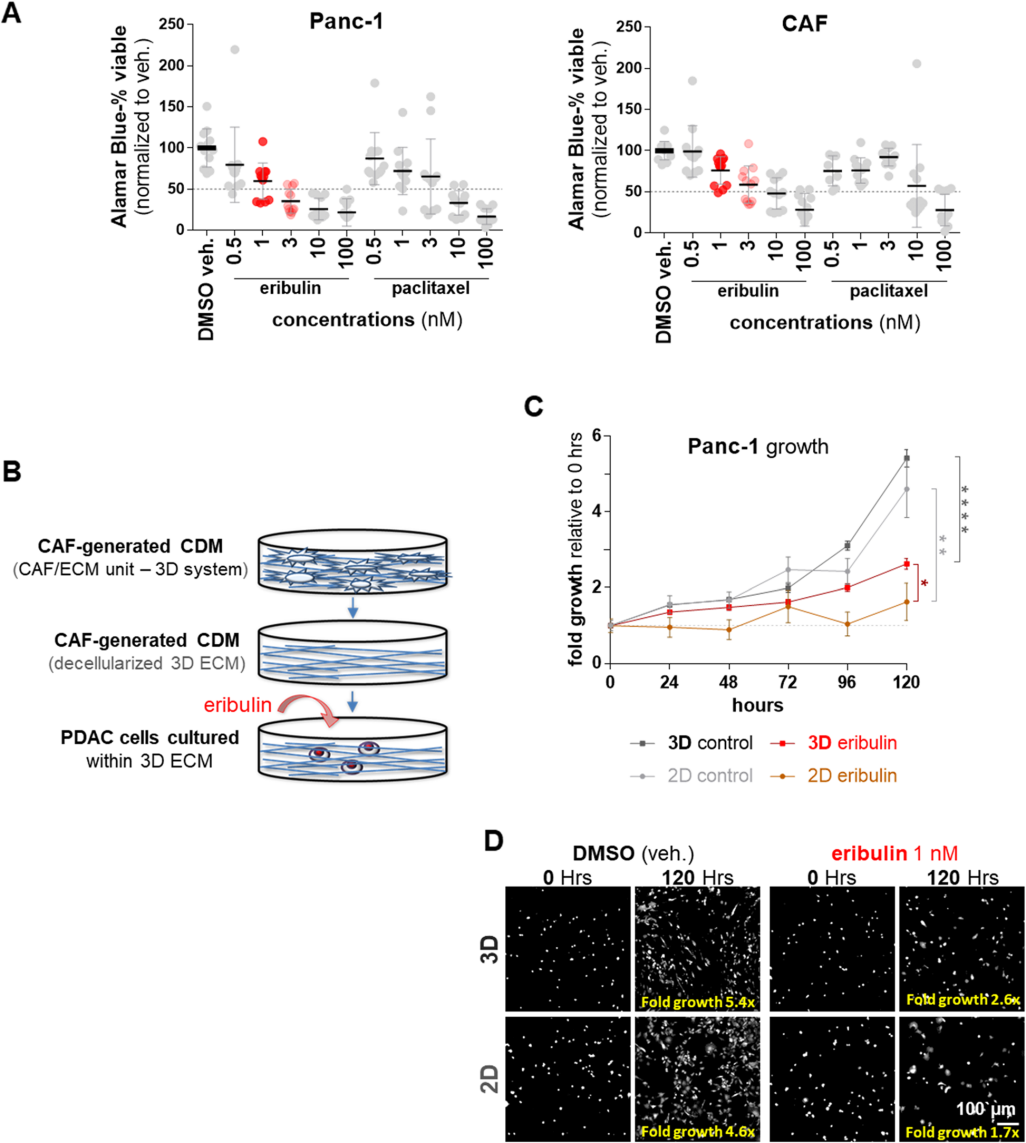
CAF-generated ECM protects PDAC cells from eribulin-induced growth inhibition. **A** Growth effects imparted by eribulin vs. paclitaxel on Panc-1 vs. CAFs, measured via Alamar Blue assay, using classical 2D culturing conditions. Graphs show the percentage of viable cells, compared to DMSO vehicle control. Drugs were used at the stated concentrations for 5 days. Experiments were repeated 4 times using a minimum of 4 replicates per experimental condition. **B** Illustration depicting experimental conditions whereby RFP-expressing Panc-1 cells cultured within decellularized CAF-generated CDMs (blue) were treated daily with eribulin (red). **C** Graph indicating fold cell-growth values normalized to each condition at day 0 and measured every 24 h for 120 h. Values shown represent means and standard errors, while asterisks are indicative of a nonparametric t-test, used to compare between data sets at 120 h; * *P* < 0.05; ** *p* < 0.01; *** *P* < 0.001; **** *p* < 0.000. **D** Representative images of Panc-1 cell area coverages at baseline (time 0) and 120 h, using 2D and 3D conditions showing DMSO vehicle and eribulin treatments. Fold area coverage increases are listed in yellow as fold growth. Scale bar corresponds to 100 μm (white)

For statistics in all experiments, we used the one-way ANOVA test to compare between conditions; all asterisks indicate comparisons of specified conditions vs. DMSO treatment (* *p* < 0.05, ** *p* < 0.01, *** *p* < 0.001, **** *p* < 0.0001) unless otherwise indicated in the figure legend. Graph Pad Prism 7.0 software (San Diego, California) was used for all analyses. For comparison of fold growth rates in Fig. 1 C, a nonparametric t-test was used to compare between each pair data set at 120 h. Note that for all (2D and 3D) culture conditions, effects of DMSO (e.g., vehicle) were used to normalize results and were compared to the behavior of untreated cells, finding no significant differences, unless otherwise noted.

## Results

### CAF-generated CDM protects PDAC cells from eribulin induced growth inhibition

Pre-clinical and clinical studies have demonstrated that CAF elimination could accelerate PDAC tumorigenesis [7–9]. Hence, in this study, it was initially important to assure that the eribulin needed half-maximal inhibitory concentration (IC_50_), intended to eliminate PDAC cells, was not significantly harmful to human PDAC CAF/ECM units. To test this, CAFs harvested from surgical samples [34] and the well-known PDAC cell line Panc-1 [36], were independently cultured, using classic two-dimensional (2D) conditions, in the presence of increased concentrations of eribulin. Results were compared to results obtained with paclitaxel, as this drug constitutes another anti-microtubule drug often used in the clinic, while DMSO served as vehicle control. Figure 1 A shows that 5-day treatment with 1 nM and 3 nM eribulin rendered a 40% and 65% decline (mean 60% ± 22% and 35% ± 15%, respectively) in PDAC cell viability compared to vehicle control (DMSO mean 100% ± 23%). Interestingly, CAFs failed to reach 50% cell growth inhibition in the presence of eribulin at the same concentrations (76% ± 17% for 1 nM and 59% ± 23% for 3 nM) (Fig. 1 A).

It is important to note that the in vivo-mimetic three-dimensional (3D) CDM system is suitable for measuring ECM modulation of human cancer cell growth in response to chemotherapeutic drugs [30]. Therefore, we next questioned whether the rate of PDAC growth inhibition induced by eribulin could be altered in the presence of decellularized CAF-generated CDMs. For this, PDAC cell growth was gauged under classic 2D conditions, or within decellularized CAF-generated 3D CDMs (Fig. 1B). Cells were treated daily with 1 nM eribulin or equal volumes of DMSO vehicle (i.e., control). PDAC cell growth values were calculated as area coverages, compared to baseline (e.g., day 0), every 24 h for a total of 120 h (e.g., 5 days). Results showed that PDAC cells grow at similar rates under both 2D and 3D conditions; area coverages measured in time presented with fit value linear regression slopes of about 0.03 for both 2D and 3D DMSO-treated cells (2.6 × 10^− 2^ ± 6.0 × 10^− 3^ and 3.2 × 10^− 2^ ± 7.9 10^− 3^, respectively). As expected, the slope value obtained for PDAC cells treated with eribulin under classic 2D conditions was the lowest (4.8 × 10^− 3^ ± 2.5 × 10^− 3^), indicating that the 2D condition resulted in the highest inhibition of PDAC cell growth in response to eribulin. Notably, the decellularized CAF-generated 3D CDMs system (e.g., 3D substrate) provided a level of protection from eribulin with a growth slope of about 0.02 (± 1.7 × 10^− 3^), which is 2.5-fold higher than its 2D counterpart (Fig. 1 C). Further, comparisons of initial PDAC cell area coverage at baseline vs. 120 h, indicated that PDAC cells achieved ~ 5-fold growth increases when treated with DMSO. Yet, area coverage of eribulin-treated PDAC cells cultured under 2D conditions failed to double after 5 days (1.7 fold), while the same cells cultured within the 3D CDMs increased the area coverage by almost 3-fold (Fig. 1 C-D). Altogether, these data indicate that decellularized ECMs, obtained using our CAF-generated CDM system, protect cancer cells from eribulin-imparted growth inhibition.

### Eribulin-treated CAFs simulate selected traits of TGFβ-deficient CAFs

**Fig. 2:**
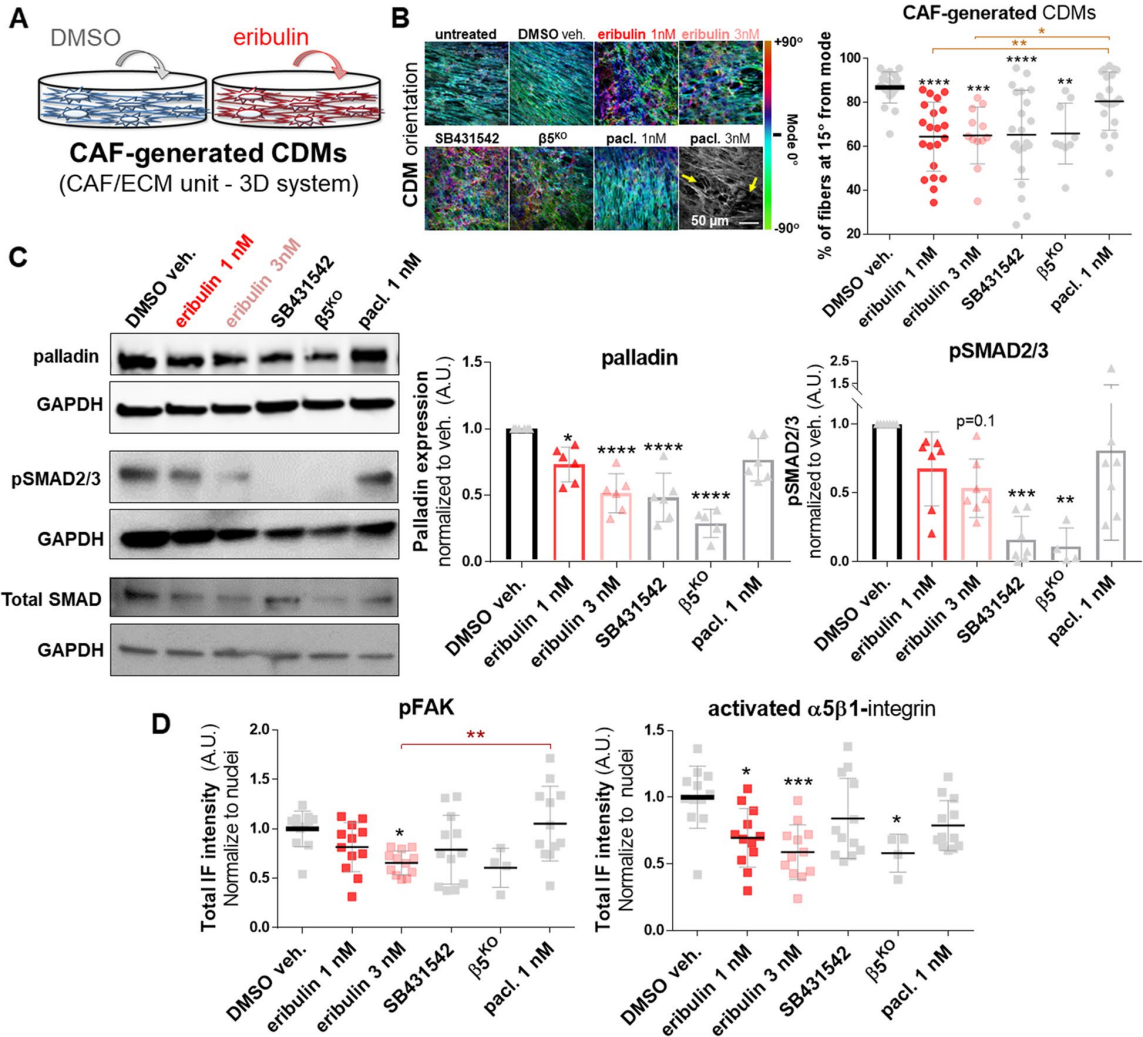
Eribulin normalizes CAF traits in the CDM system. **A** Illustration depicting the experimental conditions for CAF-generated CDMs treated with DMSO (blue) or eribulin (red) during matrix production. **B** Representative images of CAF-generated CDMs that resulted from the assorted experimental conditions used during the 5-day matrix production. Images show fibronectin fibers detected by indirect immunofluorescence and digitally pseudo-colored to depict the orientation angles of ECM fibers. The colored bar on the right represents the angle distributions and was normalized to show mode angle as 0° (cyan). The scale bar in the bottom right image corresponds to 50 μm. Note that CAFs treated with paclitaxel at 3 nM were not quantified due to the lack of fibrillogenesis as indicated by the yellow arrows pointing to ECM gaps and by the low cellularity and ECM thickness, measured below 7 μm, as shown in accompanying Supplemental Fig. 1 A. The graph indicates the levels of fiber alignment, quantified as the percentage of fibers oriented within 15° from the mode angle. **C** Representative cropped immunoblots (for full matching blots see Supplemental Fig. 2) of lysates collected at the end of CAF-generated CDM production showing representative bands for palladin, phospho-SMAD2/3 (pSMAD2/3), and total SMAD2/3 (SMAD), using GAPDH as an intracellular protein loading control. Accompanying graphs depict levels of palladin and activated SMAD (pSMAD2/3) gauged from immunoblots and normalized to levels attained under DMSO treated conditions. **D** The total intensities of phospho-FAK (pFAK) and activated α5β1-integrin, calculated using the MetaMorph software 7.8.0.0 and measuring total intensities with the same inclusive intensity thresholds set for all conditions, were normalized to cell numbers (nuclei counts). Experiments were repeated 4 times while a minimum of 6 images were acquired per sample. For statistical significance, one-way ANOVA was used to compare to DMSO vehicle. * *P* < 0.05; ** *p* < 0.01; *** *P* < 0.001; **** *p* < 0.0001

It is well known that fibroblastic cell traits and functions, in vivo and in vitro, are modulated and sustained by the self-generated interstitial ECM [37]. Hence, the above-listed results prompted to question whether the ECM-imparted protection against eribulin could be overcome using this drug to treat CAFs during ECM production (Fig. 2A). We previously reported that blocking the TGFβ receptor, TGFR1, modulates CAF-generated CDM production. This is because TGFR1 inhibition renders a disorganized (e.g., isotropic) ECM with “normalized” features, which significantly diverges from the highly aligned (e.g., anisotropic) matrix fibers produced by intact tumor-supportive CAFs [15, 27]. Of note, it has been reported that in addition to acting as a microtubule dynamics-blocker [38], eribulin limits levels of pSMAD2/3, which are indicative of obstructing canonical TGFβ signaling [20, 39]. The eribulin-modulated CAF/ECM unit features were hence compared to results attained with paclitaxel and with the small molecule SB431542, a well-known TGFR1 inhibitor. In addition, the reported αvβ5-integrin deficient (β5^KO^) CAFs, known to perpetuate normalized CAF/ECM unit features (and functions) akin to TGFβ inhibition [15], served as a positive control indicative of “normalized” CAF/ECM units. To assure a high quality of ECM production via the CDM system, controls included measuring cell density and ECM thickness. Respectively, these tests assure that the 5-day lasting treatments do not significantly alter CAF numbers, or the ability of CAFs to uphold fibrillogenesis (Supplemental Fig. 1A). Note that solely sufficiently thick ECM samples were used to assess fiber alignment (Fig. 2B and Supplemental Fig. 1B). To this end, the “normalized” β5^KO^ CAF/ECM unit control exhibited both an expected modestly-low cell density [27], and a productive fibrillogenesis [15] (Supplemental Fig. 1A). Similarly, results revealed that eribulin does not compromise ECM fibrillogenesis (Supplemental Fig. 1A). CAFs treated with 3 nM paclitaxel attained poor fibrillogenesis, which invalidated the further assessment of these (Fig. 2B and Supplemental Fig. 1A). Regarding ECM alignment, results showed that eribulin significantly reduced the parallel fiber organization of CAF-generated CDMs, suggesting a CAF/ECM unit “normalization” (Fig. 2B and Supplemental 1B). Further, these isotropic fiber disorganization levels were comparable to the levels obtained with SB431542, as well as in ECM fibers produced by the β5^KO^ CAFs (Fig. 2B and Supplemental Fig. 1B). For rigor purposes, CAF’, the CRISPR/Cas9 control of the β5^KO^ CAFs, as well as an additional PDAC patient-harvested CAF line (CAF 2) and a lung cancer CAF line were also tested, and all rendered similar eribulin-modulated changes in CAF-generated CDM fiber alignment (Supplemental Fig. 1B). Together these results suggest that eribulin effectively phenocopies the effects attained with TGFβ deficient CAFs (e.g., using SB431542 treatment or in β5^KO^ CAFs) during ECM fibrillogenesis. Alternatively, treatment with paclitaxel either fails to alter ECM alignment, or if used at higher concentrations (e.g., 3 nM) compromises CAF fibrillogenesis (Fig. 2B and Supplemental Fig. 1 A).

In addition to the effects of eribulin on ECM alignment, we questioned whether this drug could hinder known intracellular CAF biomarkers indicative of tumor-supportive CAF function [40]. For this, we quantified the levels of expression of the actin-bundling protein palladin, as well as constitutive SMAD2/3 activity (e.g., levels of pSMAD2/3). The two above-listed biomarkers are known to be upregulated in tumor-supportive PDAC CAFs in response to TGFβ, and their intracellular fibroblastic levels inversely correlate with PDAC patient overall survival [15, 22, 41]. Similarly to using SB431542 or β5^KO^ CAF/ECM units, increased doses of eribulin (e.g., 1 nM and 3 nM) triggered a modest 30% and 50% decrease in palladin expression (0.73 ± 0.13 and 0.51 ± 0.14) respectively, with analogous yet more noticeable tendencies for pSMAD2/3 (0.67 ± 0.27 and 0.53 ± 0.21), compared to vehicle control (Fig. 2 C, Supplemental Figs. 1C and 2). Endogenous levels of total SMAD2/3 were also queried (Fig. 2 C and Supplemental Fig. 2 A-C). Further, levels of focal adhesion kinase tyrosine-397 phosphorylation (pFAK), as well as intensities of the active conformation of the fibronectin receptor α5β1-integrin were also assessed. Note that these two markers are also indicative of tumor-supportive CAF function in vitro (e.g., in the 3D CDM system), and have been associated with worse patient outcomes in vivo [15, 40]. Although levels of pFAK solely showed a modest tendency of 20% reduction in response to 1 nM eribulin (81% ± 25%), a significant decrease of 34% (66% ± 12%) in the activity of this kinase was evident with 3 nM eribulin (Fig. 2D). Similarly, constitutive levels of active α5β1-integrin decreased by 30% and 40% in CAF-generating CDMs treated with 1 nM and 3 nM eribulin, respectively (Fig. 2D). Note that all tendencies were similar to results obtained with SB431542 and in β5^KO^ CAF/ECM units, yet paclitaxel did not affect the gauged pFAK or active α5β1-integrin levels.

In addition to the CAF functional biomarkers listed above, we also tested whether lipid droplet accumulation, indicative of resident pancreatic fibroblast normalization [15], constitutes another feature of eribulin treatment. For this CAF/ECM units were treated as before and the levels of lipid droplets accumulated by the cells were measured using Nile Red. Excitingly, eribulin treatment prompted a 26% (± 0.13) increase in Nile Red positive droplets with similar increase tendencies noted in CAF/ECM units treated with SB431542 (13% ± 0.05 increase), and in β5^KO^ CAF/ECM units (16% ± 0.13), compared to DMSO (Supplemental Fig. 3). Another function of tumor supportive CAFs is the secretion of pro-tumoral cytokines. Intriguingly, while eribulin-treated CAF/ECM units showed a noticeable decrease in IL6 levels, which indeed simulated SB431542 treatment, eribulin also prompted an unexpected increase in IL8 and TGFβ (Supplemental Fig. 4).

In order to ask whether eribulin could overcome activation by TGFβ, we treated β5^KO^ CAF/ECM units with TGFβ in the presence or the absence of eribulin or SB431542. Results shown in Supplemental Fig. 5, suggested that similarly to SB431542, eribulin prevents the myofibroblastic activation of these cells. This was noted by the ability of eribulin to counteract the TGFβ-induced collapse, indicative of fibroblastic cell/ECM unit contraction. Altogether, these data suggest that eribulin triggers a phenotypic transition from tumor-supportive to tumor-suppressive, which is akin to known TGFβ inhibition for most but not all the tested traits, thus rendering CAF/ECM units with selected features reminiscent of “normalized” CAFs [15, 40].

### Eribulin treated CAFs generate CDMs deficient in prompting a normal-to-CAF transition

**Fig. 3:**
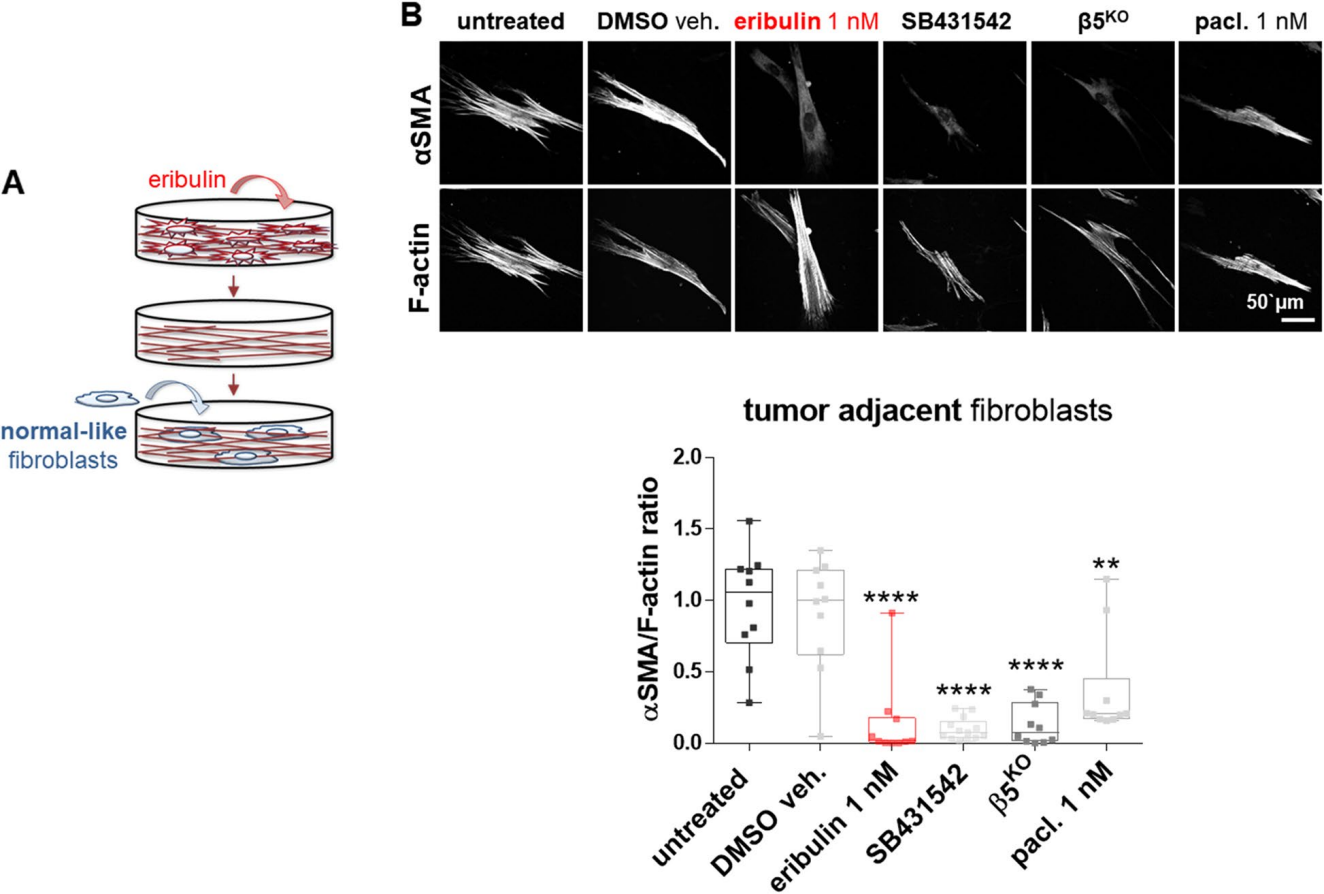
CAFs treated with eribulin generate ECMs that fail to induce a normal-to-CAF transition. **A** Illustration showing naïve fibroblasts re-plated into decellularized CDMs obtained from eribulin-treated CAFs. **B** Representative confocal immunofluorescent images showing monochromatic reconstitutions of αSMA and stress fibers (F-actin). Accompanying box and whiskers graph representing ratios of αSMA localization at F-actin positive pixels (error bars = maximum and minimum values while all median measured values are shown as individual squares). For statistical significance, ordinary one-way ANOVA was used comparing all data to the DMSO veh. control. * *P* < 0.05; ** *p* < 0.01; *** *P* < 0.001; **** *p* < 0.0001

Normal fibroblasts respond to CAF-generated CDMs by undergoing a phenotypic switch, analogous to CAF activation [27]. Since CDMs produced by eribulin-treated CAFs showed “normalized” features (e.g., isotropic/disorganized ECM fibers), we questioned whether these matrices have lost the functional ability to prompt a normal-to-CAF transition. For this, we used fibroblasts harvested from benign tissue adjacent to matching PDAC and seeded them onto decellularized CDMs (Fig. 3A). Notably, decellularized CDMs obtained from eribulin-treated CAFs were no longer able to induce normal-to-CAF activation (Fig. 3B), as denoted by the amount of αSMA localized to stress fibers [15, 27]. Although some basal αSMA levels were expressed by tumor-adjacent fibroblastic cells, the eribulin-treated CAF-generated decellularized CDMs solely induced about 2% of this protein to localize at stress fibers (median = 0.016 ± 0.28), compared to levels prompted by CDMs generated from CAFs treated with DMSO. These profiles were similar to the ones reported for CDMs obtained from SB431542- treated or β5^KO^ CAFs, which CDMs too failed to induce CAF activation [15] (Fig. 3B). Paclitaxel-treated CAFs, however, generated CDMs that were able to guide an αSMA stress fiber localization 10-fold higher than the one observed with eribulin (median = 0.2 ± 0.36) (Fig. 3B). Altogether, results suggested that eribulin treatment of CAFs during CDM production renders matrices deficient in inducing a classic normal-to-CAF activation.

### Eribulin treated CAFs generate CDMs deficient in promoting PDAC invasion and survival

**Fig. 4:**
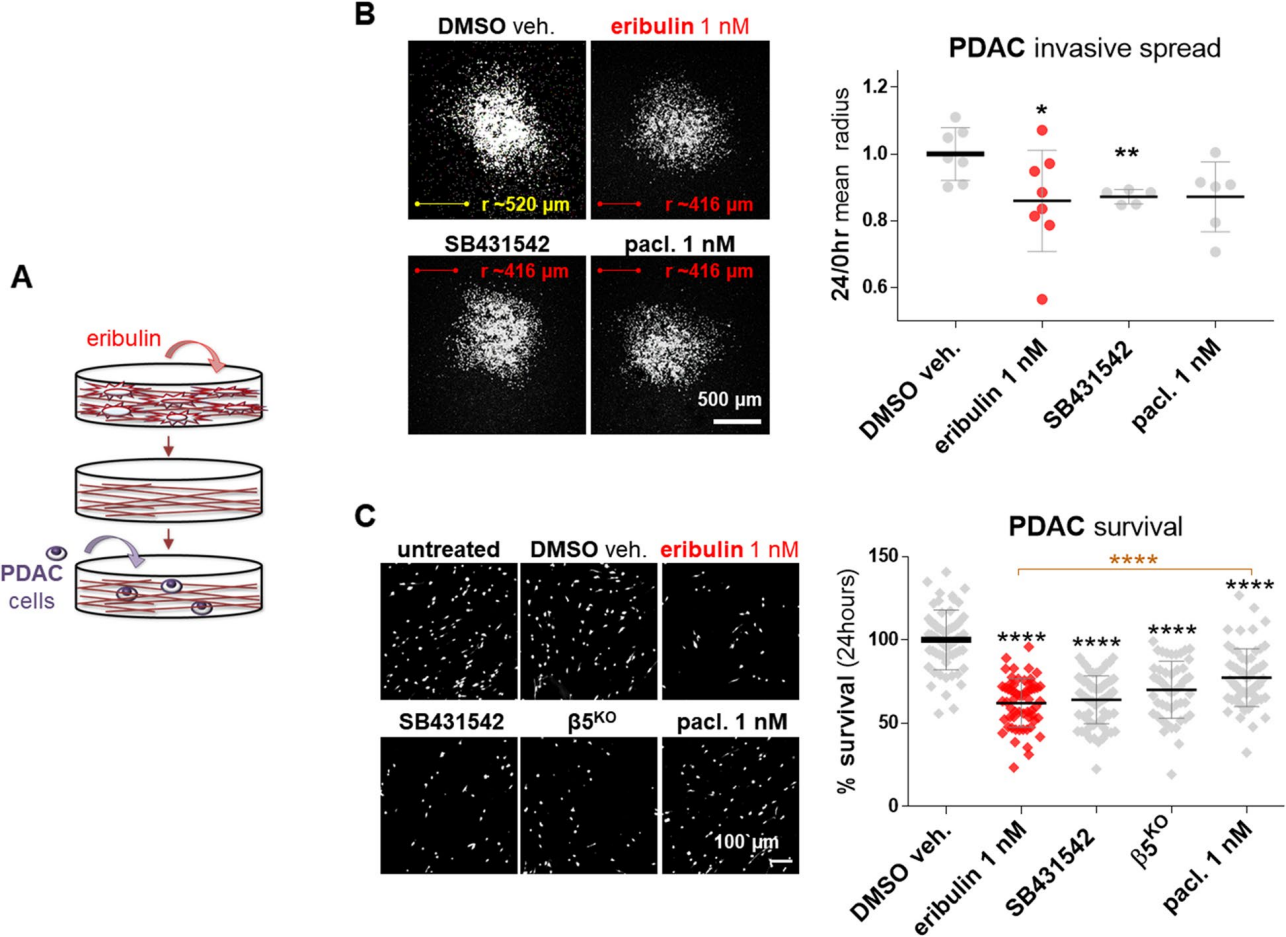
Eribulin-treated CAFs generate tumor-suppressive ECMs. **A** Illustration showing cancer cells re-plated onto decellularized CDMs obtained from eribulin-treated CAFs. **B** Representative 4x microscopy images of RFP-expressing Panc-1 spheroids that were allowed to spread for 24 h (using nutrient-avid conditions) onto CDMs generated by CAFs treated with DMSO, eribulin, SB431542, or paclitaxel. Scale bar corresponds to 500 μm (white), while average spheroid spread radiuses (r) are shown in yellow for control and red for experimental conditions. The corresponding graph, on the right, shows 24 h/0hr ratios of measured mean-radiuses. For statistical significance, the Mann-Whitney test was used to compare each condition to the DMSO veh. control. * *P* < 0.05; ** *p* < 0.01; *** *P* < 0.001; **** *p* < 0.0001. **C** RFP-expressing Panc-1 cells were cultured into the assorted listed decellularized CDMs, as in B, only this time using nutrient-deprived conditions. Viable cells were gauged following 24, 48, and 72 h and representative 24-hour images are shown, while 48 and 72 h are in Supplemental Fig. 6. Scale bar corresponds to 100 μm. The graph indicates the percentage of survival, represented by RFP-expressing Panc-1 cells. Area coverage obtained data were normalized to DMSO veh., which average was set as 100% survival. For statistical significance, a one-way ANOVA (Turkey’s multiple comparisons) test was used to compare all conditions. Black asterisks denote comparisons to DMSO, while the eribulin vs. paclitaxel comparison is noted in orange. * *P* < 0.05; ** *p* < 0.01; *** *P* < 0.001; **** *p* < 0.0001

We previously reported that similar to CDMs produced by normal fibroblasts, TGFβ-deficient CAFs (e.g., SB431542-treated and β5^KO^ CAFs) render CDMs that are compromised in promoting PDAC cell invasive spread and in sustaining PDAC cell survival under nutrient-deprived conditions [22, 23, 35]. Hence, we next questioned whether eribulin-treated CAFs would generate CDMs that too fail to sustain these pro-PDAC cell functions (Fig. 4 A). Using decellularized CDMs as before, results showed that when PDAC spheroids were cultured within CDMs produced by eribulin-treated CAFs for 24 h, the invasive spreading capacity of the cancer cells was reduced by 20%, compared to vehicle-treated CAF-generated CDM controls (Fig. 4B).

Importantly, PDAC cell survival assessed under nutrient-deprived conditions at 24, 48, and 72 h, was significantly compromised (Fig. 4 C and Supplemental Fig. 6). After only 24 h of culturing cells within CDMs generated by eribulin-treated CAFs, PDAC survival was decreased by 38% (± 14.6%) compared to the vehicle-treated control. Of note, these survival levels were also significantly limited by CDMs generated from SB431542-treated (36% ± 14.4%) and β5^KO^ (30% ± 17.1%) CAFs. Paclitaxel-treated CAFs generated CDMs that were solely able to modestly limit PDAC cell survival (23% ± 17.4%; Fig. 4 C). These results demonstrate that CAFs treated with eribulin produced CDMs incapable of providing a nutritional benefit to starved PDAC cells. Taken together, these data suggest that eribulin treatment effectively hinders CAFs’ ability to produce pro-tumoral CDMs, similar to matrices derived from normal-like fibroblasts [23].

### Eribulin fails to revert the survival benefit provided by intact CAF-generated CDMs

**Fig. 5:**
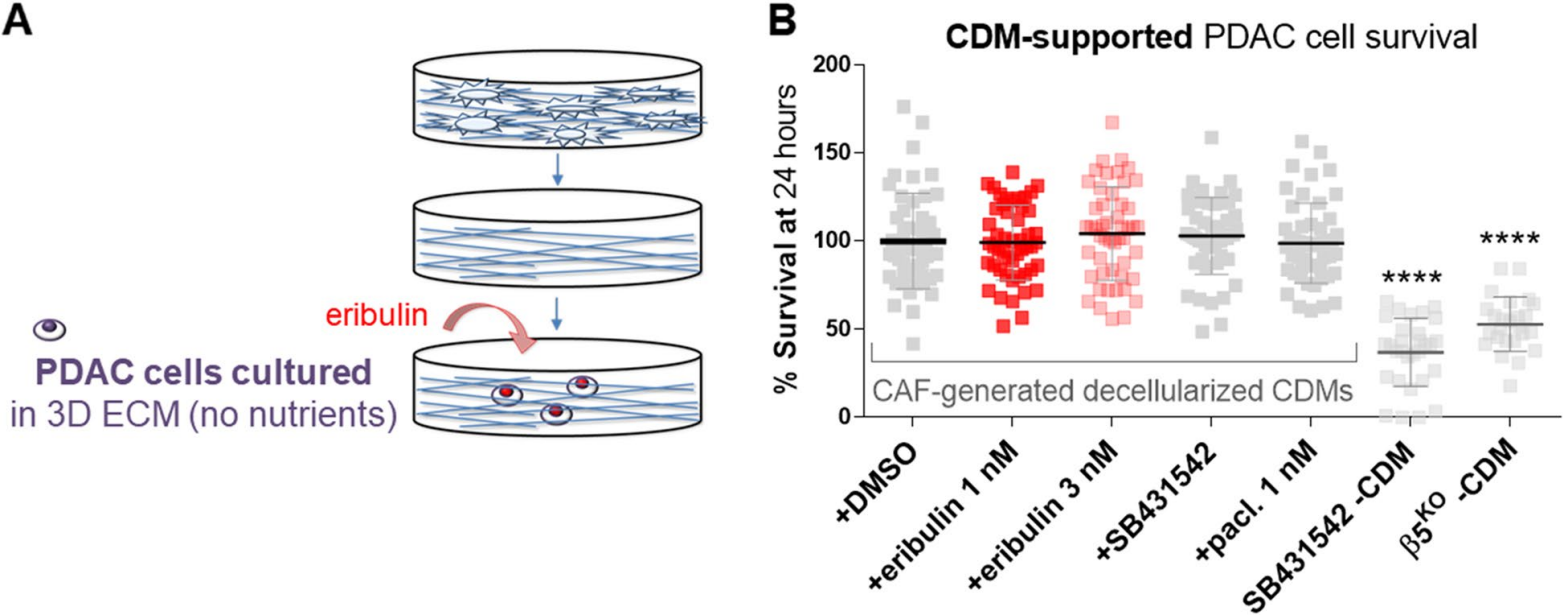
Eribulin fails to reverse the survival benefit provided by CAF ECMs. **A** Illustration showing the used experimental conditions whereby eribulin was added to nutrient-deprived cancer cells cultured within intact CAF-generated decellularized CDMs. **B** The graph shows the survival percentage of RFP-expressing Panc-1 cells treated with DMSO, eribulin, SB431542, or paclitaxel cultured for 24 h in intact CAF-generated CDMs. CDMs obtained from SB431542 treated and β5^KO^ CAFs (as in Fig. 4C) served as “normalized” CDM controls. Data were normalized to DMSO veh., which average was set as 100% survival. For statistical significance, a one-way ANOVA (Turkey’s multiple comparisons) test was used to compare all conditions. Black asterisks denote comparisons to DMSO, while the eribulin vs. paclitaxel comparison is noted in orange. * *P* < 0.05; ** *p* < 0.01; *** *P* < 0.001; **** *p* < 0.0001

Results above (Fig. 4 C and Supplemental Fig. 6) demonstrated that CAFs treated with eribulin produced CDMs that limited PDAC cell survival under nutrient-deprived conditions. Hence, we were interested to test whether eribulin could also revert the benefit imparted by intact CAF-generated CDMs in sustaining PDAC cell survival under the same nutrient-poor conditions. For this, PDAC cells were cultured for 24 h within decellularized but otherwise intact CAF-generated CDMs under nutrient-deprived conditions and treated with either eribulin, SB431542, paclitaxel, or a DMSO (Fig. 5 A). Results were compared to survival levels obtained using known tumor-suppressive CDMs (e.g., generated by SB431542-treated or β5^KO^ CAFs, as the ones used in Fig. 4 C). Of note, eribulin treatment failed to revert the protective effect imparted by decellularized CDMs (Fig. 5B). Likewise, neither SB431542 nor paclitaxel treatments hindered this CDM pro-survival benefit. These results suggest that eribulin effects on PDAC survival (e.g., Fig. 4 C and Supplemental Fig. 6), are the result of the eribulin-imparted effects on CAF/ECM units, as opposed to a direct effect upon PDAC cells. Altogether these observations are akin to reported TGFβ-deficient CAFs, whereby TGFβ signaling was shown to be uniquely necessary to produce a tumor-supportive ECM [15, 22, 33, 40].

## Discussion

In addition to being used as a third-line treatment option for patients with metastatic breast cancer [42], it was reported that eribulin prompts an overall survival benefit in a sarcoma Phase III clinical trial [43]. Importantly, several advanced PDAC patients treated with eribulin presented with a significant period (~ nine months) of stable disease [44]. This achievement is remarkable for this patient population, and therefore it justified the further investigation of using eribulin in this disease [18, 44]. These types of studies displayed encouraging efficacy for eribulin treatment in cancers that are fibrous (e.g., sarcomas) or that typically encompass a dense fibrous desmoplastic TME (e.g., advanced breast and PDAC). The above-stated facts highlight that in addition to its known microtubule-fixing function recognized for targeting epithelial cancer cells, eribulin also effectively regulates fibrous tumors like sarcoma. Further, eribulin mesylate was reported to be both a non-taxane microtubule dynamics blocker [38], as well as an EMT inhibitor that reinstitutes epithelial characteristics to mesenchymal cells via limiting SMAD activity [20]. Of note, SMAD activation lies downstream to TGFR1 canonical signaling, induced by the activated/mature TGFβ ligand [45]. It is hence not surprising that eribulin was shown to rapidly inhibit TGFβ-induced signaling [39]. Consequently, in this study, we hypothesized that eribulin could affect fibroblastic cells, which also greatly depend on TGFβ signaling for their activation [46]. The goal of this study was hence to assess the in vitro modulating effects that eribulin imparts on PDAC CAF function. For this, we used our well-characterized in vivo-mimetic desmoplastic system, referred to in this study as the CAF-generated CDM system, which is ideally suited for testing phenotypes and functions of human CAF/ECM units [15, 23, 40].

By incorporating human CAFs with CAF-generated ECM, the above-mentioned CDM system was previously developed to simulate human desmoplasia in vitro [28, 34, 40]. The CDM is a pathophysiologic-relevant system that has been successfully used in numerous human cohorts [15, 23, 47]. The desmoplastic/CAF CDM system serves for studying drug effects on cancer regulated by the TME [30, 31, 48], as well as for measuring the direct influences of drugs on modulating fibroblastic cell function [22, 23, 29, 33]. Since quiescent/normal fibroblasts are naturally tumor-suppressive [10] and because CAF elimination has been demonstrated to be advantageous to PDAC progression and deleterious to patients [7–9], the field has placed significant efforts on identifying potential stroma-normalizing drugs that can harness the natural tumor-suppressive function of fibroblastic cells [6, 12, 13].

To study the potential of drugs to impart a normalizing function upon CAF/ECM units, an important control to incorporate is a bona fide “normalized” CAF cell line. To this end, β5^KO^ CAF/ECM units constitute an ideal human cellular model as these functional units present with a phenotype and function akin to perpetuating TGFβ inhibition [15]. In fact, β5^KO^ CAFs have been reported to generate CDMs with tumor-suppressive functions and traits [15, 22, 33]. Using β5^KO^ CAFs, we previously demonstrated that canonical TGFβ signaling is necessary for the production of tumor-supportive ECMs. However, once these matrices are produced, normal fibroblast undergo tumor-suppressive to tumor-supportive activation in response to these pro-tumoral natural scaffolds/substrates in a TGFβ independent manner [15]. The main difference between pharmacologically TGFβ-inhibited CAF/ECM and β5^KO^ CAF/ECM units is that the latter perpetuate a tumor-suppressive “normalized” CAF/ECM unit phenotype and function, while the former is transient.

While it is well accepted that TGFβ inhibition effectively blocks pro-tumor CAF activation, anti-tumor immunity, and EMT [15, 24, 26, 49], drugs targeting TGFβ have not been very successful in clinical trials [16]. For example, based on a query of the Clinicaltrials.gov data-base, 5 trials have proposed to test the use of TGFβ inhibitory agents in gastrointestinal cancers, including in pancreatic cancer patients. Of these, one was terminated early due to a treatment-related death (NCT03451773), another reported that the tolerated dose had no noted clinical benefit (NCT00844064), a third trial was completed in early 2022, and despite promising pe-clinical data, it has not yet been published. Nonetheless, some data was shared at an international meeting, yet solely renal and lung, but not pancreatic cancer patients, were discussed (NCT02947165). Unfortunately, the remaining two trials are yet to initiate recruitment or in the means of doing so. With this in mind and considering that the level of eribulin toxicities is well tolerated in patients [18, 50], and knowing that eribulin limits canonical TGFβ signaling [20, 39], we posited that eribulin acts as a stroma-normalizing molecule that could simulate some known effects attained with TGFβ inhibitors. Results obtained from this study showed that eribulin causes increased CAF ECM fiber isotropy, as well as a modest decreased in palladin expression while limiting the constitutive activity of FAK, SMAD2/3, and α5β1-integrin. Since the above-listed traits are classically allied to tumor-supportive myofibroblastic features [40], we also queried whether inflammatory CAF features were altered by eribulin. Interestingly, we observed that similarly to TGFβ inhibition, IL6 secretion was effectively downregulated in response to eribulin. Nonetheless, while limiting TGFβ activity diminished the secreted levels of IL8 and TGFβ, these two tumor-promoting cytokines were increased in response to eribulin suggesting that some but not all TGFβ inhibiting traits are shared by SB431542 and eribulin. Altogether, the response of CAF/ECM units to eribulin suggests a tendency for inducing a tumor-supportive to tumor-suppressive CAF transition that encompasses all measured pro-tumoral myofibroblastic and some, but not all, inflammatory features [15, 22, 27, 40]. Moreover, CAF-generated CDMs produced in the presence of eribulin also lose their ability to induce CAF activation (e.g., failed to promote the reported αSMA localization to stress fibers [15, 27]). Importantly, while CAF-generated CDMs imparted modest protection against eribulin upon PDAC cells, eribulin treatment effectively “normalized” CAF-generate CDMs, which fail to support invasive spread as well as to effectively nurture PDAC cells under nutrient-deprived conditions [22, 23, 33, 35].

Despite using our well-established in vivo-mimetic fibroblastic CDM system in which we culture fibroblastic cells and their self-generated ECMs as functional units [15, 23, 27, 37], it is important to note the limitations of this study in order to avoid over interpretations. We therefore recognize that the lack of animal models in this study constitutes a noteworthy limitation. Of note, the use of animal models in studies subsequent to this work, could begin to address the numerous possible multicellular cross talk and functional interactions that take place between fibroblastic cells, other TME cells, and cancer cells in response to eribulin. Notably, a recent study carried out single cell transcription analyses, using a plethora of cancerous organs samples (including pancreatic), and concluded that fibroblastic cells are the cell type that interacts the most with other cells in the TME [51]. Therefore, future studies using eribulin in vivo could render information regarding CAF/ECM unit effects upon, for example, immune cell function. We hence advise readers to consider the below-stated conclusions solely within the context of the human in vivo-like pancreatic CAF/ECM units.

## Conclusion

**Fig. 6:**
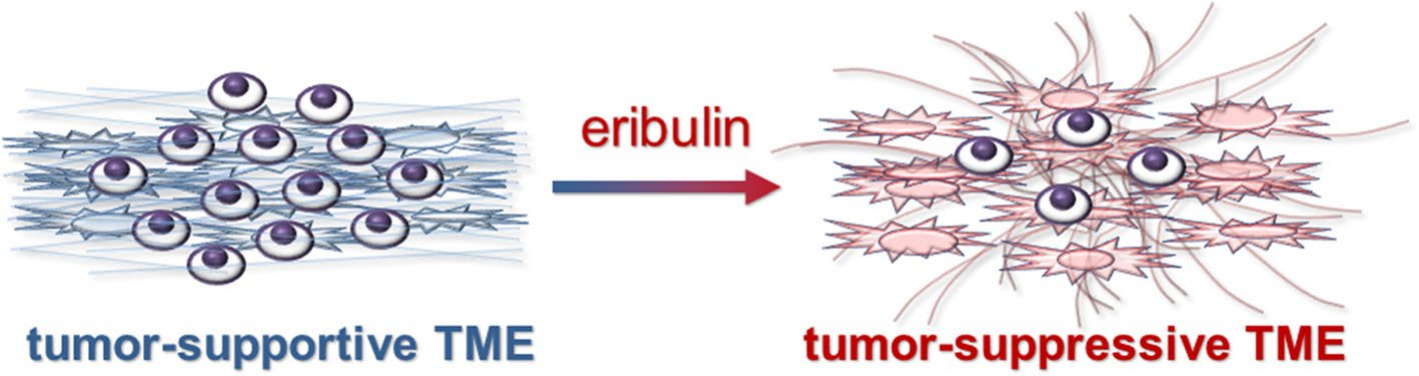
Schematic representation illustrating the tumor-suppressive effects of eribulin through normalization of CAFs. Illustration showing a tumor-permissive microenvironment (blue) and indicating that eribulin modulates CAFs in a way that these cells are prompted to generate a normalized, tumor-suppressive (red), TME

Data generated in this study suggest that eribulin simulates selected aspects of TGFβ inhibition by rendering CAFs with tumor-suppressive traits and functions. These results shed some light in potentially explaining why using eribulin in specifically fibrous-relevant cancers has been clinically successful. Further, since eribulin somewhat simulates TGFβ downregulation [24, 25], which has been shown to reinstitute anti-tumor immunity [26], results from this study also support the potential notion of combining eribulin with immune-regulatory drugs in the future. Importantly, since clinical trials that have aimed to eliminate CAF/ECM units have failed in the clinic, the TME field is now actively aiming to finding means for “normalizing” CAF/ECM units and harness their natural tumor suppressive function while exclusively targeting the tumor supportive functions. To this end, this study proposes that eribulin could serve as a “stroma normalizing” agent that renders CAF/ECM units with tumor-suppressive features and functions (Fig. 6).

## Supplementary Information

**Additional file 1:** **Supplemental Figure 1.** Eribulin normalizes CAF/ECM unit traits. (**A**) cellularity (left), measured as nucleiarea coverage per image, and ECM thickness (right) are shown for the assorted  conditions. (**B**) Graphs obtained from matricesgenerated by three independent CAFs, cultured as in Figure 2, indicating levelsof fiber alignment, quantified as the percentage of fibers oriented within 15° fromthe mode angle. (**C**) Quantifications obtained from immunoblots of lysatescollected at the end of matrix production, as in Figure 2, using an additional humanpancreatic CAF, indicating levels of palladin and pSMAD2/3 (GAPDH was againused as intracellular protein loading control). For statistical significance,one-way ANOVA was used to compare to DMSO vehicle. * *P* < 0.05; ** *p* < 0.01;*** *P* <0.001; **** *p* < 0.0001. **Supplemental Figure 2.** Original scanned films used in mainFigure 2C for palladin, pSMAD2/3 and total SMAD blots. (**A**) Panelcorresponding to the cropped portions of the immunoblots shown in **B** and **C**.Color dotted areas denote the cropped portions (and tilted orientations) thatwere used in Figure 2C. Blue for palladin, red for pSMAD2/3, and magenta fortotal SMAD. Note that the corresponding GAPDH for each blot is marked with thecorresponding dotted line color. (**B**) Blots were generated using Licor and (**C**)were obtained from scanning films that were generated with ECL. **SupplementalFigure 3.** Eribulin increases thelevels of lipid droplets in CAF/ECM units. Representative monochromaticimages of nile red staining, indicative of lipid droplets, in eribulin- or SB431542-treatedCAF/ECM units. DMSO veh. and β5^KO^CAF units were used as controls.Graph shows the fluorescent intensity of nile red positive areas normalized toHoechst (nuclei). Experiments were repeated 2 times in triplicates. Forstatistical significance, the unpaired t test with Welch’s correction was usedto compare each data set to DMSO veh; ** *p*< 0.01. **Supplemental Figure 4. **Changes in cytokine secretion in response to eribulin. ELISAs wereperformed using conditioned media collected at the end of CAF/ECM units’ matrixproduction. Measurements were read at 450 nm with reference wavelength at 570nm using Tecan Spark^TM^ 10Mmicroplate reader. Graphs indicate the concentrations of IL6, IL8 and TGFβ that were measuredfrom the assorted listed experimental conditions. Conditioned media werecollected and three technical replicates (IL6 and IL8), or two technicalreplicates (TGFβ) were performed for experiments that wererepeated two times. For statistical significance, ordinary one-way ANOVA wasused. * *P* < 0.05; ** *p* < 0.01; *** *P* < 0.001; **** *P* <0.0001. **Supplemental Figure 5. **Eribulinreduces the tension caused by addition of TGFβ to β5^KO^CAF/ECM units. Representative images of β5^KO^CAF/ECMunits treated with DMSO vehicle only (top), or with TGFβ at 10 ng/mL (middle)or 1 ng/mL (bottom), with or without eribulin at 1 nM or SB431542 are shown.Two different areas of the same condition are shown for units treated with TGFβ (10 ng/mL or 1ng/mL) to show the detachment of the CAF/ECM units (detached) and areas of thefew cells that remained attached (persistent). Two independent experiments intriplicates for each condition were conducted and the exact same phenomenon wasobserved. Images were obtained using 10x (unlabeled bars = 400 µm) or 4x (1000µm bar) via the Evos cell imaging system. **Supplemental Figure 6.** Eribulin-treated CAFs generate ECMs fail tosustain PDAC  cell survival undernutritional stress. RFP-expressing Panc-1 cells from Figure 4C  were cultured into the assorted ECMs using nutrient-depletedconditions. Viable cells  were gaugedfollowing 48, and 72 hours as area coverage. Data were normalized to  DMSO veh., which average was set as 100%survival. For statistical significance, a one-  way ANOVA (Turkey’s multiple comparisons) testwas used to compare all conditions.  Black asterisks denote comparisons to DMSO, whilethe eribulin vs paclitaxel comparison  isnoted in orange.  * *P* < 0.05; ** *p* <0.01; *** *P* <0.001; **** *p* < 0.0001.

## Data Availability

The datasets used and/or analyzed during the current study are all included in this manuscript.

## Abbreviations

αSMA: alpha smooth muscle actin
β5^KO^: β5-integrin deficient
CAFs: cancer-associated fibroblasts
CDM: cell-derived extracellular matrix
DMEM: Dulbecco’s Modified Eagle Medium
ECM: extracellular matrix
EMT: epithelial-to-mesenchymal transition
FBS: Fetal Bovine Serum
pFAK: focal adhesion kinase tyrosine-397 phosphorylation
PDAC: pancreatic ductal adenocarcinoma
pSMAD2/3: phosphorylated SMAD2/3; Small Worm Phenotype and Mothers Against Decapentaplegic 2 and 3
PVDF: polyvinylidene fluoride
RFP: red fluorescent protein
SB431542: inhibitor of TGFβ Type 1 receptor kinases
SMAD: Small Worm Phenotype and Mothers Against Decapentaplegic
TGFβ: transforming growth factor-beta
TME: tumor microenvironment
