# Supplementary material for: Eribulin normalizes pancreatic cancer-associated fibroblasts by simulating selected features of TGFβ inhibition
Source: BMC Cancer. 2022 Dec 2;22:1255. doi: 10.1186/s12885-022-10330-y (PMC9719234; doi:10.1186/s12885-022-10330-y)
Supplement: Supplementary file 1 — Additional file 1: Supplemental Figure 1. Eribulin normalizes CAF/ECM unit traits. (A) cellularity (left), measured as nucleiarea coverage per image, and ECM thickness (right) are shown for the assorted conditions. (B) Graphs obtained from matricesgenerated by three independent CAFs, cultured as in Figure 2, indicating levelsof fiber alignment, quantified as the percentage of fibers oriented within 15° fromthe mode angle. (C) Quantifications obtained from immunoblots of lysatescollected at the end of matrix production, as in Figure 2, using an additional humanpancreatic CAF, indicating levels of palladin and pSMAD2/3 (GAPDH was againused as intracellular protein loading control). For statistical significance,one-way ANOVA was used to compare to DMSO vehicle. * P < 0.05; ** p < 0.01;*** P <0.001; **** p < 0.0001. Supplemental Figure 2. Original scanned films used in mainFigure 2C for palladin, pSMAD2/3 and total SMAD blots. (A) Panelcorresponding to the cropped portions of the immunoblots shown in B and C.Color dotted areas denote the cropped portions (and tilted orientations) thatwere used in Figure 2C. Blue for palladin, red for pSMAD2/3, and magenta fortotal SMAD. Note that the corresponding GAPDH for each blot is marked with thecorresponding dotted line color. (B) Blots were generated using Licor and (C)were obtained from scanning films that were generated with ECL. SupplementalFigure 3. Eribulin increases thelevels of lipid droplets in CAF/ECM units. Representative monochromaticimages of nile red staining, indicative of lipid droplets, in eribulin- or SB431542-treatedCAF/ECM units. DMSO veh. and β5KOCAF units were used as controls.Graph shows the fluorescent intensity of nile red positive areas normalized toHoechst (nuclei). Experiments were repeated 2 times in triplicates. Forstatistical significance, the unpaired t test with Welch’s correction was usedto compare each data set to DMSO veh; ** p< 0.01. Supplemental Figure 4. Changes in cytokine secretion in resp [file 12885_2022_10330_MOESM1_ESM.pptx]

## Slide 1
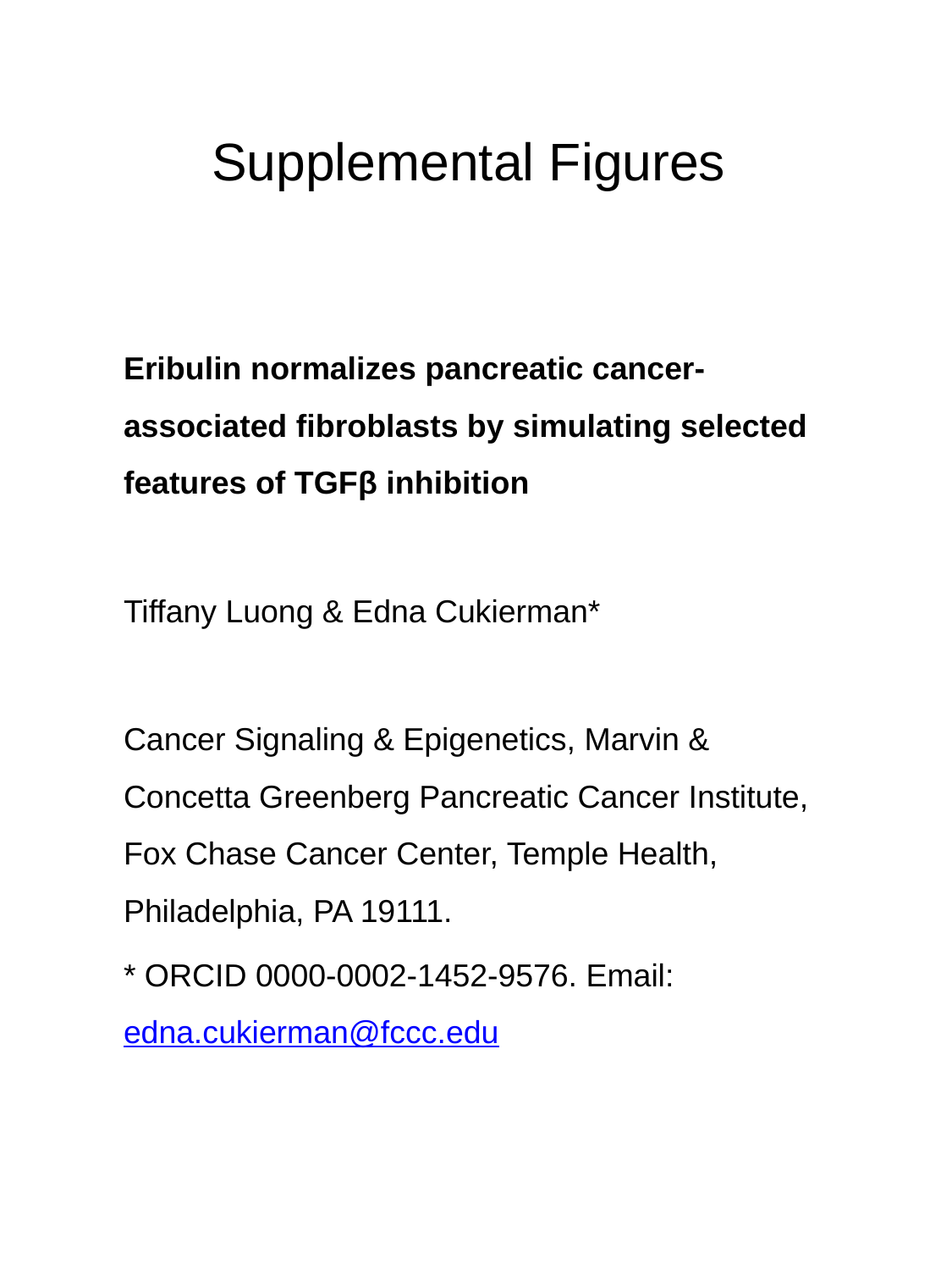

Supplemental Figures
Eribulin normalizes pancreatic cancer-associated fibroblasts by simulating selected features of TGFβ inhibition
Tiffany Luong & Edna Cukierman*
Cancer Signaling & Epigenetics, Marvin & Concetta Greenberg Pancreatic Cancer Institute, Fox Chase Cancer Center, Temple Health, Philadelphia, PA 19111.
* ORCID 0000-0002-1452-9576. Email: edna.cukierman@fccc.edu

## Slide 2
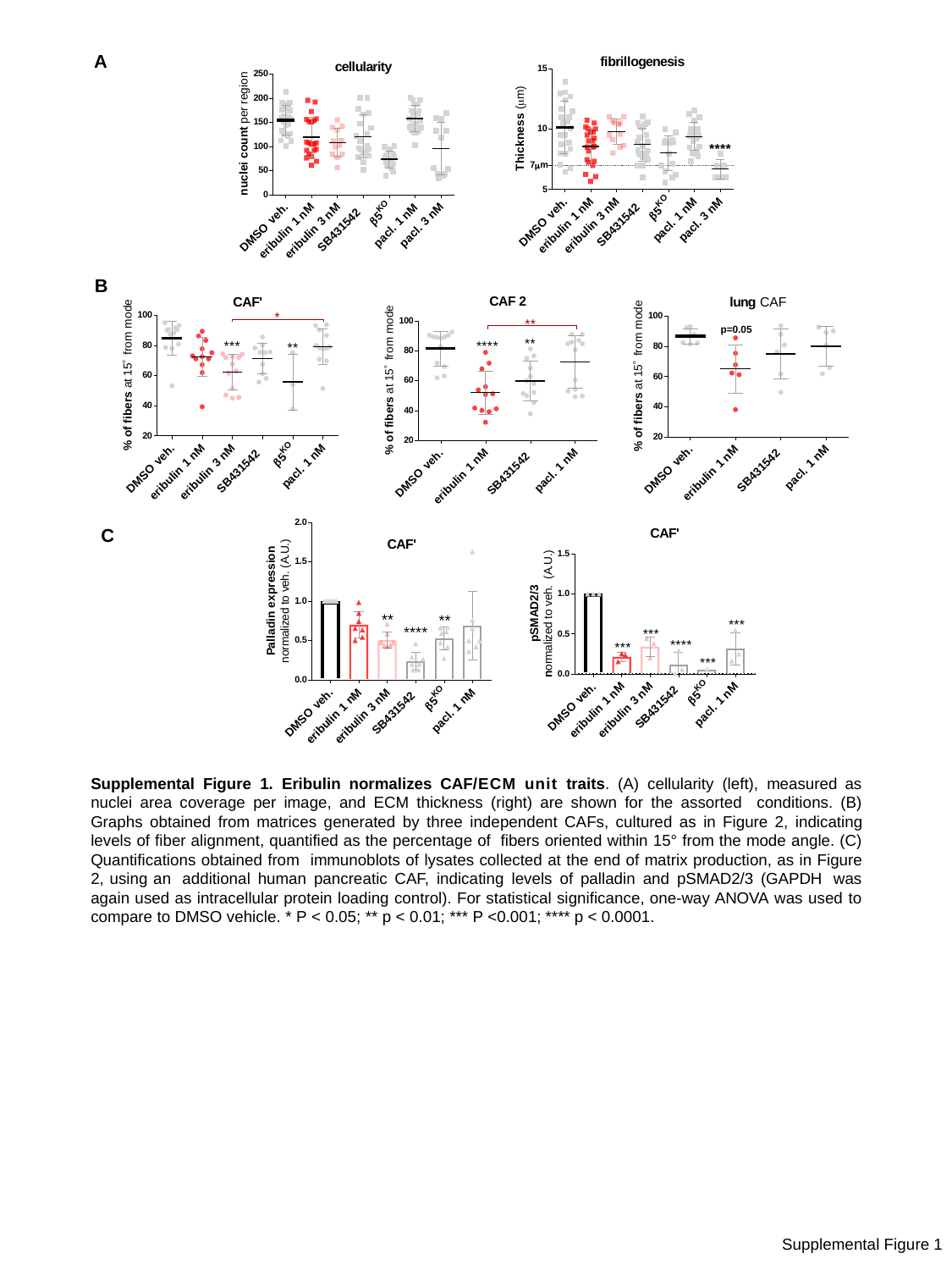

A
B
C
Supplemental Figure 1. Eribulin normalizes CAF/ECM unit traits. (A) cellularity (left), measured as nuclei area coverage per image, and ECM thickness (right) are shown for the assorted conditions. (B) Graphs obtained from matrices generated by three independent CAFs, cultured as in Figure 2, indicating levels of fiber alignment, quantified as the percentage of fibers oriented within 15° from the mode angle. (C) Quantifications obtained from immunoblots of lysates collected at the end of matrix production, as in Figure 2, using an additional human pancreatic CAF, indicating levels of palladin and pSMAD2/3 (GAPDH was again used as intracellular protein loading control). For statistical significance, one-way ANOVA was used to compare to DMSO vehicle. * P < 0.05; ** p < 0.01; *** P <0.001; **** p < 0.0001.
Supplemental Figure 1

## Slide 3
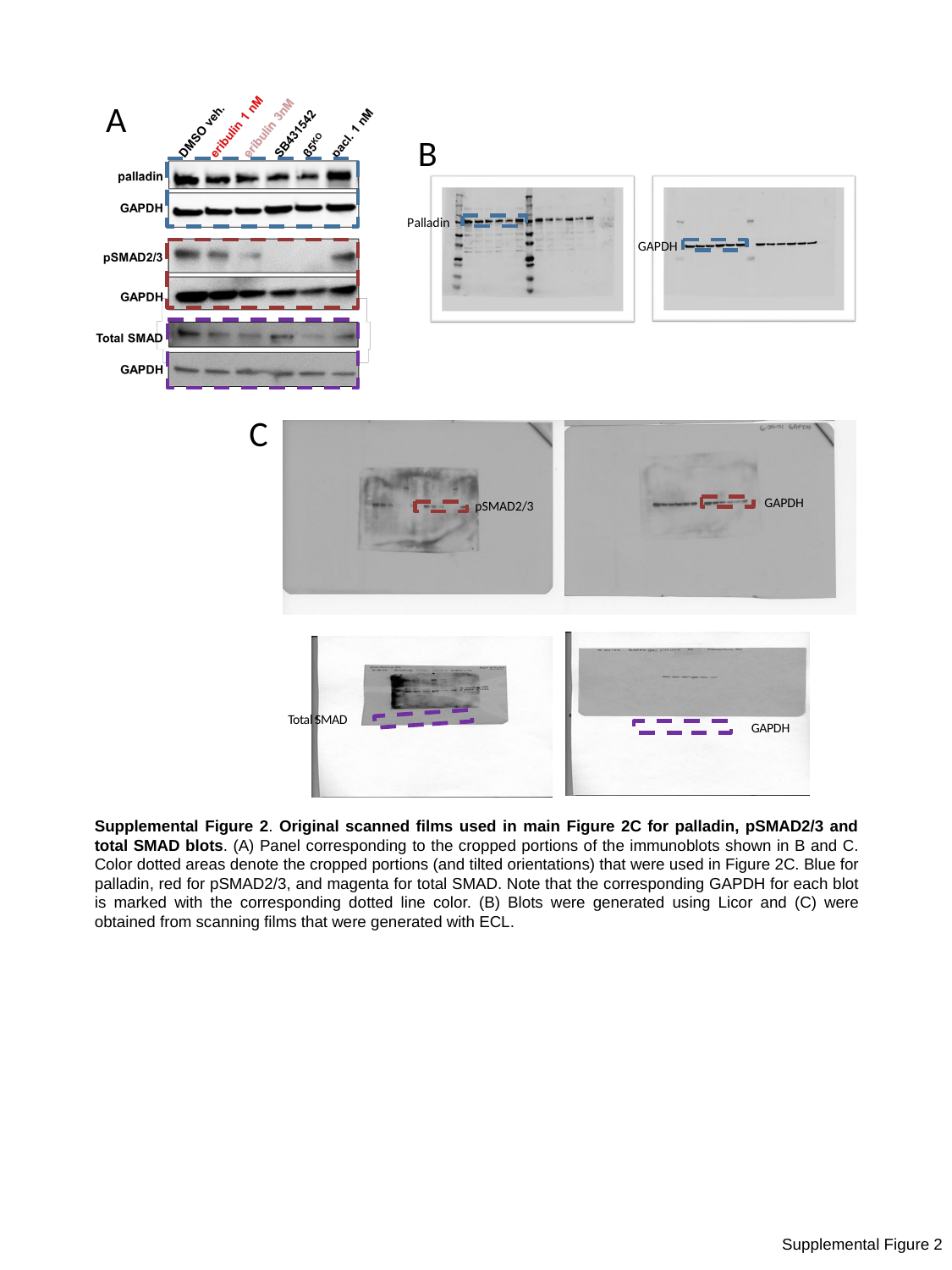

A
B
# Palladin
GAPDH
C
GAPDH
pSMAD2/3
Total SMAD
GAPDH
Supplemental Figure 2. Original scanned films used in main Figure 2C for palladin, pSMAD2/3 and total SMAD blots. (A) Panel corresponding to the cropped portions of the immunoblots shown in B and C. Color dotted areas denote the cropped portions (and tilted orientations) that were used in Figure 2C. Blue for palladin, red for pSMAD2/3, and magenta for total SMAD. Note that the corresponding GAPDH for each blot is marked with the corresponding dotted line color. (B) Blots were generated using Licor and (C) were obtained from scanning films that were generated with ECL.
Supplemental Figure 2

## Slide 4
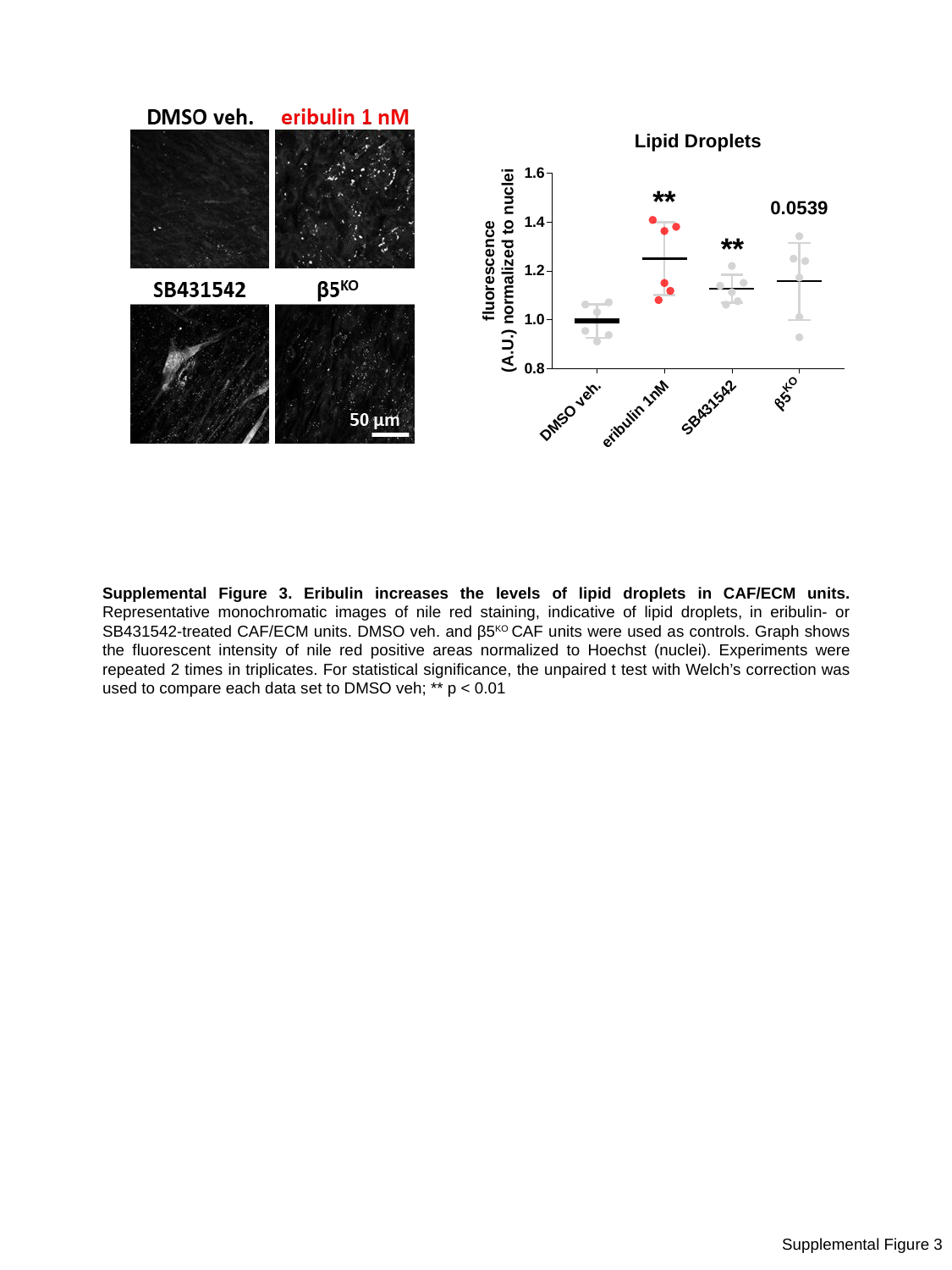

Supplemental Figure 3. Eribulin increases the levels of lipid droplets in CAF/ECM units. Representative monochromatic images of nile red staining, indicative of lipid droplets, in eribulin- or SB431542-treated CAF/ECM units. DMSO veh. and β5KO CAF units were used as controls. Graph shows the fluorescent intensity of nile red positive areas normalized to Hoechst (nuclei). Experiments were repeated 2 times in triplicates. For statistical significance, the unpaired t test with Welch’s correction was used to compare each data set to DMSO veh; ** p < 0.01
50μm
Supplemental Figure 3

## Slide 5
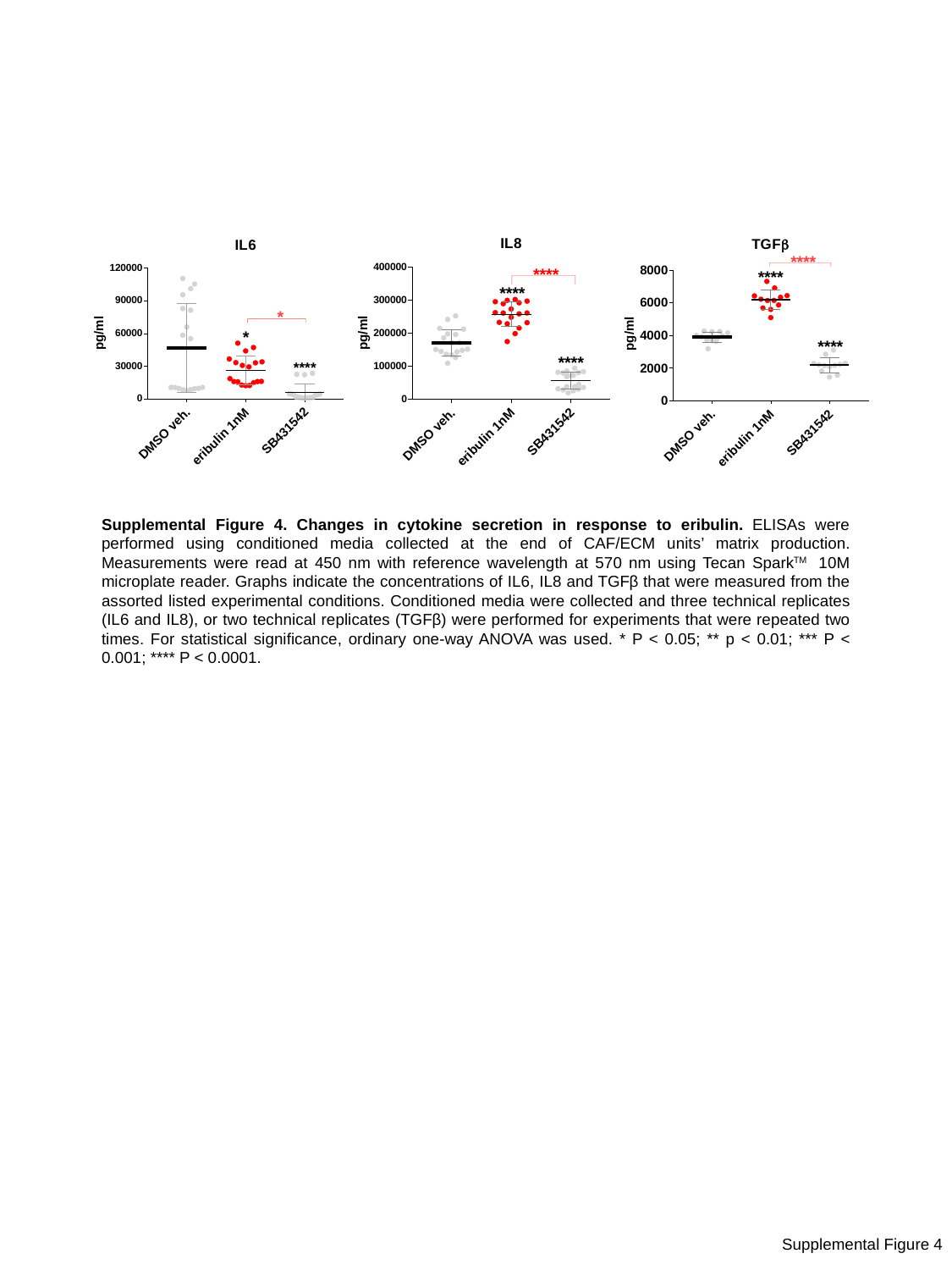

Supplemental Figure 4. Changes in cytokine secretion in response to eribulin. ELISAs were performed using conditioned media collected at the end of CAF/ECM units’ matrix production. Measurements were read at 450 nm with reference wavelength at 570 nm using Tecan SparkTM 10M microplate reader. Graphs indicate the concentrations of IL6, IL8 and TGFβ that were measured from the assorted listed experimental conditions. Conditioned media were collected and three technical replicates (IL6 and IL8), or two technical replicates (TGFβ) were performed for experiments that were repeated two times. For statistical significance, ordinary one-way ANOVA was used. * P < 0.05; ** p < 0.01; *** P < 0.001; **** P < 0.0001.
Supplemental Figure 4

## Slide 6
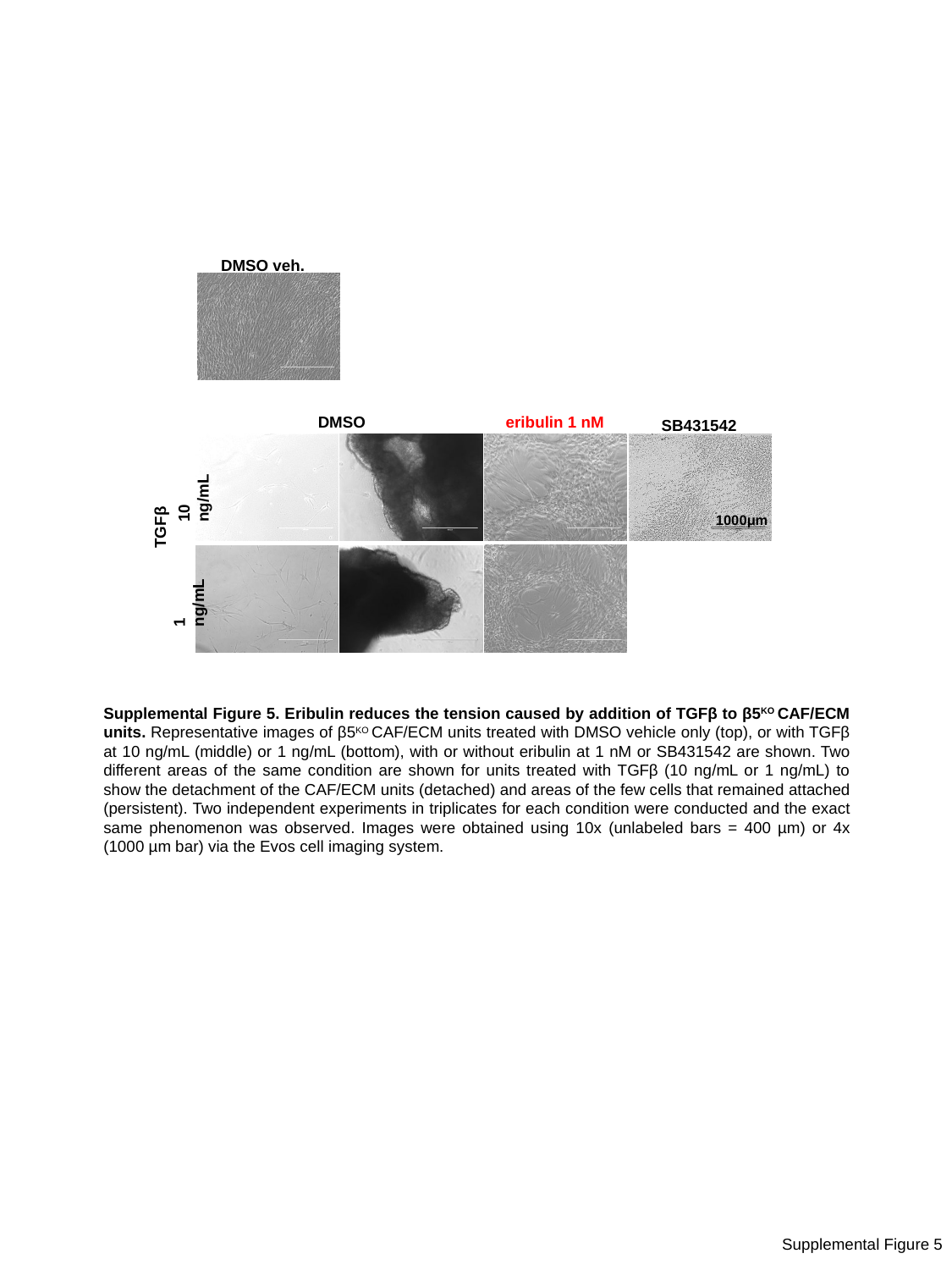

DMSO veh.
DMSO
eribulin 1 nM
SB431542
10 ng/mL
TGFβ
1000μm
1 ng/mL
Supplemental Figure 5. Eribulin reduces the tension caused by addition of TGFβ to β5KO CAF/ECM units. Representative images of β5KO CAF/ECM units treated with DMSO vehicle only (top), or with TGFβ at 10 ng/mL (middle) or 1 ng/mL (bottom), with or without eribulin at 1 nM or SB431542 are shown. Two different areas of the same condition are shown for units treated with TGFβ (10 ng/mL or 1 ng/mL) to show the detachment of the CAF/ECM units (detached) and areas of the few cells that remained attached (persistent). Two independent experiments in triplicates for each condition were conducted and the exact same phenomenon was observed. Images were obtained using 10x (unlabeled bars = 400 µm) or 4x (1000 µm bar) via the Evos cell imaging system.
Supplemental Figure 5

## Slide 7
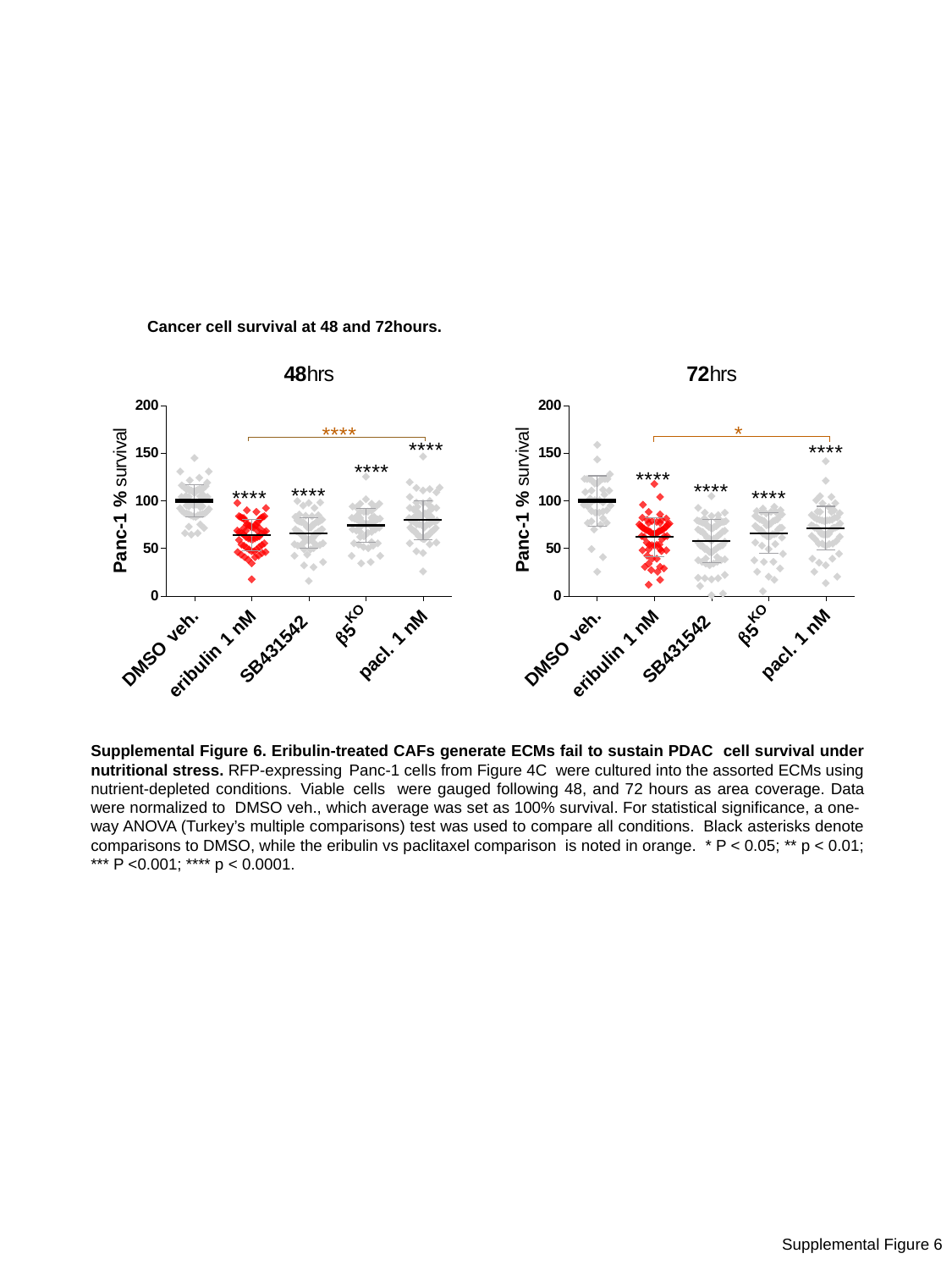

Cancer cell survival at 48 and 72hours.
Supplemental Figure 6. Eribulin-treated CAFs generate ECMs fail to sustain PDAC cell survival under nutritional stress. RFP-expressing Panc-1 cells from Figure 4C were cultured into the assorted ECMs using nutrient-depleted conditions. Viable cells were gauged following 48, and 72 hours as area coverage. Data were normalized to DMSO veh., which average was set as 100% survival. For statistical significance, a one- way ANOVA (Turkey’s multiple comparisons) test was used to compare all conditions. Black asterisks denote comparisons to DMSO, while the eribulin vs paclitaxel comparison is noted in orange. * P < 0.05; ** p < 0.01; *** P <0.001; **** p < 0.0001.
Supplemental Figure 6
